# Discriminative motif discovery in DNA and protein sequences using the DEME algorithm

**DOI:** 10.1186/1471-2105-8-385

**Published:** 2007-10-15

**Authors:** Emma Redhead, Timothy L Bailey

**Affiliations:** 1 Institute for Molecular Bioscience, University of Queensland, Brisbane, Qld. 4072 Australia

## Abstract

**Background:**

Motif discovery aims to detect short, highly conserved patterns in a collection of unaligned DNA or protein sequences. Discriminative motif finding algorithms aim to increase the sensitivity and selectivity of motif discovery by utilizing a second set of sequences, and searching only for patterns that can differentiate the two sets of sequences. Potential applications of discriminative motif discovery include discovering transcription factor binding site motifs in ChIP-chip data and finding protein motifs involved in thermal stability using sets of orthologous proteins from thermophilic and mesophilic organisms.

**Results:**

We describe DEME, a discriminative motif discovery algorithm for use with protein and DNA sequences. Input to DEME is two sets of sequences; a "positive" set and a "negative" set. DEME represents motifs using a probabilistic model, and uses a novel combination of global and local search to find the motif that optimally discriminates between the two sets of sequences. DEME is unique among discriminative motif finders in that it uses an informative Bayesian prior on protein motif columns, allowing it to incorporate prior knowledge of residue characteristics. We also introduce four, synthetic, discriminative motif discovery problems that are designed for evaluating discriminative motif finders in various biologically motivated contexts. We test DEME using these synthetic problems and on two biological problems: finding yeast transcription factor binding motifs in ChIP-chip data, and finding motifs that discriminate between groups of thermophilic and mesophilic orthologous proteins.

**Conclusion:**

Using artificial data, we show that DEME is more effective than a non-discriminative approach when there are "decoy" motifs or when a variant of the motif is present in the "negative" sequences. With real data, we show that DEME is as good, but not better than non-discriminative algorithms at discovering yeast transcription factor binding motifs. We also show that DEME can find highly informative thermal-stability protein motifs. Binaries for the stand-alone program DEME is free for academic use and is available at

## Background

Sequence motif discovery has been applied to discover many types of patterns in DNA and amino acid sequences. For example, motif discovery has been used extensively to elucidate putative transcription factor binding sites [1,2] and to discover protein-protein interaction domains [3]. In most cases, motif discovery algorithms take as input only a set of sequences hypothesized to contain a biologically important sequence pattern, and search for patterns that are unlikely to occur by chance. Usually, the concept of "occurring by chance" is captured in some kind of probabilistic model of "random" sequences. Since motifs are usually short and can be highly variable sequence patterns [1], a challenging problem for motif discovery algorithms is to distinguish functional motifs from random patterns that are over-represented by chance.

Discriminative motif discovery attempts to find motifs that occur more frequently in one set of sequences compared to another set. This can help with the problem of distinguishing functional motifs from randomly occurring sequence patterns, because the negative set of sequences may be a better representation of "random" sequences than can easily be captured in a probabilistic model. For example, in order to discover the TFBS motif of a transcription factor (TF), the set of DNA probes from a ChIP-chip [4-6] or DIP-chip [7] experiment that do *not *bind to the TF can be used as the negative sequence set. The actual TFBSs may be more strongly over-represented in the DNA probes that do bind the TF when compared to a negative set of non-binding probe sequences than when compared to a random model of DNA.

Another natural application for discriminative motif discovery is in the search for differences in proteins that have evolved in different environments. Orthologs of a single bacterial protein can be divided in two sets according to whether the organism is a thermophile or a mesophile [8]. Using the thermophilic orthologs as the positive set and the mesophilic orthologs as the negative set, discriminative motif discovery can be used to find motifs that are indicative of a high-temperature environment. These motifs might differ only slightly from the corresponding sites in the mesophilic sequences [8], and may be embedded in much longer conserved domains that would be reported by a non-discriminative motif discovery algorithm.

Many algorithms have been designed to solve motif discovery problems. Most of these algorithms, including ALIGNACE [9], CONSENSUS [10], MEME [11], PATTERN-BRANCHING [12] and YMF [13], are not specifically designed for discriminative motif discovery. Some algorithms, such as WEEDER [14], do make use of a set of negative sequences in scoring candidate motifs. A few algorithms have been developed specifically for discriminative motif discovery, including ALSE [15], DIPS [16], DME [17] and SEEDSEARCH [18].

Both discriminative and non-discriminative motif discovery algorithms can be loosely grouped according to how they represent a motif. A motif may be represented as: 1) strings (or regular expressions), 2) position-specific weight matrices (PWMs), or, 3) collections of sites. String-based methods represent the motif as a sequence of letters, possibly allowing wildcards or "ambiguity characters" to represent variability in the motif. PATTERN-BRANCHING, WEEDER and YMF (non-discriminative) and SEEDSEARCH (discriminative) use a string representation. In contrast, a PWM specifies a score for each base/amino acid at each position of the motif, assuming independence between positions in the motif. When applied to DNA binding site motifs, PWMs have a strong theoretical basis relating their scores to free energy of binding [19-22]. PWMs are used by CONSENSUS and MEME (non-discriminative) and by ALSE, DIPS and DME (discriminative). The representation of a motif as a collection of sites is used by Gibbs sampling algorithms such as ALIGNACE and GLAM [23] (non-discriminative).

Several approaches have been applied to search for discriminative motifs. SEEDSEARCH [18] uses exhaustive enumeration in discrete string space to search for discriminative motifs. SEEDSEARCH counts the number of occurrences of a string, allowing a specified number of mismatches, then applies a hypergeometric significance test to discover patterns that are enriched in the positive set relative to the negative set. The enriched patterns are expanded to construct a set of PWMs and an EM-like (expectation maximization [24]) heuristic is used refine the model parameters.

DME (**D**iscriminating **M**atrix **E**numerator) [17] discovers discriminative motifs using an exhaustive, enumerative search of a discrete PWM space. That is, given a finite set of possible PWM columns, DME constructs all possible matrices of a specified width. DME applies a likelihood function to score the relative over-representation of the motif in the positive set.

DIPS (**D**iscriminative **P**WM **S**earch) [16] applies a novel probabilistic score, the "*w*-score", to represent the number and strength of PWM matches in a sequence. A novel hill-climbing heuristic is used to maximise the difference between the mean *w*-score for the positive and negative sequences. The ALSE(**AL**l **SE**quences) [15] algorithm has a very similar approach, using an EM-like refinement step for refining a PWMs and a scoring function based on the hypergeometric distribution, a distribution frequently used for modelling over-representation.

Another approach to discriminative motif discovery was taken by Segal *et al*. [25] and extended by Sharan *et al*. [26]. They use a discriminative motif finder as a component of a larger system that integrates promoter primary structure, localization and expression data to predict gene expression from sequence motifs. Their algorithm, which we will refer to as Segal-Sharan, uses a two-step process. First, it discovers discriminatory string-based motifs using SEEDSEARCH. Then, it converts these to PWMs and uses conjugate gradient [27] find PWMs that maximize a probabilistic scoring function.

The scoring function used by Segal-Sharan is based on two probabilistic sequence models, one for sequences containing a motif site, and one for sequences without a motif site. Sequences with a site are assumed to contain a single motif occurrence, and are modeled using the OOPS (One Occurrence Per Sequence) sequence model [11]. Motif occurrences are assumed to be distributed according to the PWM, treated as a position-specific frequency matrix. Sequences without a site are modelled using a 0-order Markov process. The overall data model of the Segal-Sharan algorithm has two flavors; one forcing all positive sequences to contain a site, and a variation, which we refer to here as the NOOPS (Noisy OOPS) model, allowing a fraction of the positive sequences to contain no motif occurrence [28]. In either case, negative sequences are assumed not to contain a motif site.

The Sharan-Segal algorithm labels the input sequences as "1" (positive class) or "0" (negative class). The scoring function, *F*(*D*, *θ*), is the log conditional probability of the class labels given the sequences in the dataset, *D*, and the data model parameters, *θ*. The algorithm attempts to maximize the scoring function with respect to *θ*, where

F(D,θ)=∑<X,C>∈Dlog⁡P(C|X,θ).
 MathType@MTEF@5@5@+=feaafiart1ev1aaatCvAUfKttLearuWrP9MDH5MBPbIqV92AaeXatLxBI9gBaebbnrfifHhDYfgasaacH8akY=wiFfYdH8Gipec8Eeeu0xXdbba9frFj0=OqFfea0dXdd9vqai=hGuQ8kuc9pgc9s8qqaq=dirpe0xb9q8qiLsFr0=vr0=vr0dc8meaabaqaciaacaGaaeqabaqabeGadaaakeaacqWGgbGrcqGGOaakcqWGebarcqGGSaaliiGacqWF4oqCcqGGPaqkcqGH9aqpdaaeqbqaaiGbcYgaSjabc+gaVjabcEgaNjabdcfaqjabcIcaOiabdoeadjabcYha8Hqabiab+HfayjabcYcaSiab=H7aXjabcMcaPaWcbaGaeyipaWJae4hwaGLaeiilaWIaem4qamKaeyOpa4JaeyicI4SaemiraqeabeqdcqGHris5aOGaeiOla4caaa@4C53@

Here *D *is the dataset of labeled sequences <**X**, *C *>, where *C *is the class label of the sequence **X**. The SEEDSEARCH algorithm is used to find string motifs, and these are converted to PWMs, which are used as initial estimates for *θ*. Conjugate gradient is then used to refine each initial *θ*.

In this work, we have developed a discriminative motif discovery algorithm called DEME (**D**iscriminatively **E**nhanced **M**otif **E**licitation). DEME is based on the discriminative framework of Segal *et al*. [25]. However, we apply a novel combination of global and local search to learn the parameters of the motif model that maximise the discriminative objective function (refer to Eqn. 1).

Since a string based approach has been shown to be effective for both synthetic and real motif discovery problems [1], the DEME global search algorithm searches in string space. In contrast to the hypergeometric approach of Segal *et al*. [25] and Sharan *et al*. [26], we use "substring search" [11] and "pattern branching" [12] to find good starting points for conjugate gradient. Substring search samples all substrings contained in the positive set and has been shown to work well for both DNA and protein motif discovery problems [11]. Pattern branching follows substring search to expand the search space by considering strings in the local neighbourhood of the sample strings.

To improve the search using conjugate gradient, we reparameterize the objective function to ensure that all solutions are consistent with the underlying sequence models and to allow us to use Bayesian priors on the columns of the PWM model to prevent over-fitting.

Although intended as a general purpose motif discovery algorithm, DEME includes refinements to make it more effective with protein sequences. To improve the search for protein motifs, DEME utilises prior knowledge of amino similarities to estimate the motif model parameters. That is, DEME uses the PAM120 substitution matrix and a Dirichlet mixture model to assign similar weights to amino acids with similar properties. In contrast, a simple Dirichlet prior is used for DNA sequences to prevent over-fitting. Naturally, other refinements can be imagined to improve performance in particular types of DNA or protein motif discovery problems.

A second contribution of this paper is a set of synthetic data problems for discriminative motif discovery. The synthetic problems are intended to simulate situations where algorithms such as DEME would be useful. The idea is derived from the so-called "standard challenge problem" introduced by Pevzner *et al*. [29] as a way of testing non-discriminative motif finders. The standard challenge problem specifies a synthetic DNA dataset consisting of 20 length-600 sequences, each containing an artificially generated motif occurrence. The motif is represented a string of length 15, and each occurrence contains exactly four mismatches. We augment the standard challenge problem by including a set of negative sequences and define four synthetic discriminative motif discovery problems. These include problems where a variant of the motif is planted in the negative sequences; where the negative sequences contain a strong, "decoy" motif; and where the motif is underrepresented in the negative sequences. We also describe a synthetic problem where the planted motif is generated using a PWM model based on real transcription factor PWMs. We evaluate DEME using these synthetic problems, and complement this with an evaluation of its ability to discover transcription factor binding motifs in yeast. We also illustrate the usefulness of DEME for motif discovery problems in protein sequences.

## Results and discussion

### Algorithm

Given a labeled dataset of positive and negative sequences, *D*, DEME discovers a motif of width *w *that discriminates between the two sets by maximizing the objective function given in Eqn. 1. The maximization is done over the model parameters, *θ *(described in the next section). We use a different type of global search than Sharan-Segal and apply a local search that enforces informative constraints on the model parameters.

Global search is applied first, using substring search [11] followed by "pattern branching" [12] to search for string motifs that can be used as "seeds" for local search. Seed motifs are scored using an objective function closely related to Eqn. 1. Substring and branching search convert each string ("seed") to a position-specific frequency matrix (PSFM), and use the same objective function as used for local search, Eqn. 6, to compute the score for the PSFM. The seed with the best score is used to initialize the parameters of the motif model (*θ*_*M*_) then conjugate gradient (CG) is applied to refine the model parameters.

Substring search scores every length-*w *substring in the positive dataset. A fixed-size heap is used to save the best-scoring candidate seeds. After substring search is complete, branching search expands the search space by mutating single positions in each of the seeds in the heap. One iteration of branching search scores all possible seeds that are exactly one mutation distant from a seed in the original heap. The best scoring seeds are stored in a new heap. Multiple iterations of branching search allow seeds to be found that are several mutations distant from any subsequences in the positive dataset.

Global search is complete when the last iteration of branching search is finished. Local search is now employed to refine the data model. The best scoring seed from global search is used to initialize the parameters of the motif model, *θ*_*M*_. Since the motif model contains the parameters of discrete distributions, *θ*_*M *_is mapped to a weights matrix, **W**, and conjugate gradient is run in **W **space. The logarithm of *θ*_*M *_is the mapping function that is applied as this maps *θ*_*M *_to an equivalent set of unconstrained parameters. DEME outputs the best motif found by CG, along with the sites predicted to be occurrences of the motif. The position with the highest log odds score (see Eqn. 8) in each sequence is the predicted site.

The following sections describe the data model, objective function, local search and global search in more detail.

#### Data model

DEME discovers a discriminating motif by fitting a data model to a set of labeled sequences. The data model has a set of parameters, *θ*, which consists of *θ*_*M *_(a motif model), *θ*_*B *_(a background model which describes non-motif sequence), *λ *(the probability of a positive sequence containing a motif site) and (the prior probability of a sequence being labeled positive). The input to DEME is a sequence dataset, *D*, and the desired motif width, *w*. The sequence dataset consists of a set of labeled sequences <**X**, *C *>, where **X **is a sequence, <*X*_1_, *X*_2_, ..., *X*_*L *_>, of length *L*, over the alphabet Σ, of length |Σ|. For each sequence, **X**, *C *is its class label. Sequences with *C *= 1 are referred to as "positive" sequences and sequences with *C *= 0 are "negative" sequences.

**Figure 1:**
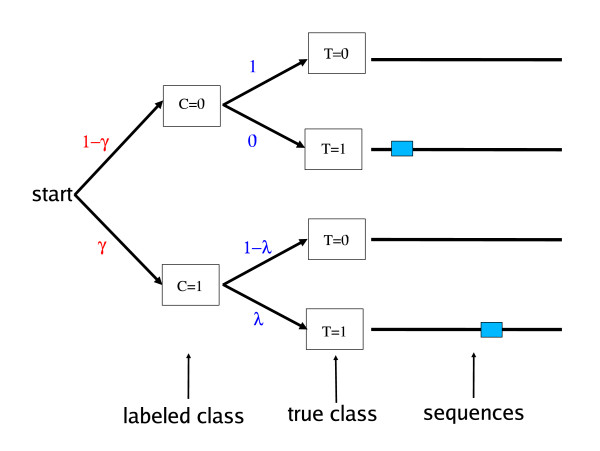
**The DEME data model**. Labels on arcs show the probabilities of choosing the labelled class, *C*, of a sequence, and the true class, *T*. When *T *= 0, sequences are generated using just the background model, *θ*_*B*_. When *T *= 1, sequences contain a motif site, generated by motif model *θ*_*M*_, inserted in random sequence generated by *θ*_*B*_.

DEME models the sequences in its input set as being generated according to the process illustrated in Fig. 1. First the labeled class, *C*, is chosen with *Pr*(*C *= 1) = *γ*. Then, the true class of the sequence, *T*, is chosen. Then the sequence is generated. If *T *= 0, a random sequence without a planted motif site is generated using a 0-order Markov process with parameter vector *θ*_*B*_, where *θ*_*B *_[*a*] is the probability of observing the letter *a *at any position in the sequence. If *T *= 1, a motif site is generated and inserted at a random position in a random sequence generated using *θ*_*B*_. The motif site is generated using a PSFM, *θ*_*M*_, whose entries, *θ*_*M*_[*a*, *i*], give the probability of observing letter *a *at position *i *in a motif site.

Negative sequences are assumed by DEME not to contain a motif site, so *Pr*(*T *= 0|*C *= 0) = 1 (see Fig. 1). Positive sequences may contain one or zero motif sites, and the probability of a positive sequence containing a site is *Pr*(*T *= 1|*C *= 1) = *γ*. If requested by the user, DEME can fix *λ *= 1, in which case all positive sequences are assumed to contain a motif site. This is referred to as the OOPS data model. By default, DEME assumes that some positive sequences may not contain a motif, and will attempt to learn the value of *λ*. This is referred to as the NOOPS data model.

For simplicity of exposition, we assume in what follows that all sequences have the same length, *L*, and we introduce variable *N *to represent the number of possible positions for a motif site, *N *= *L *- *w *+ 1. For convenience, we also define two derived variables, V and *q*. *V *specifies the ratio of the prior probabilities of a sequence containing or not containing a motif site, and is

V=P(T=1)P(T=0),=γλ1−γλ.
 MathType@MTEF@5@5@+=feaafiart1ev1aaatCvAUfKttLearuWrP9MDH5MBPbIqV92AaeXatLxBI9gBaebbnrfifHhDYfgasaacH8akY=wiFfYdH8Gipec8Eeeu0xXdbba9frFj0=OqFfea0dXdd9vqai=hGuQ8kuc9pgc9s8qqaq=dirpe0xb9q8qiLsFr0=vr0=vr0dc8meaabaqaciaacaGaaeqabaqabeGadaaakeaafaqadeGabaaabaGaemOvayLaeyypa0ZaaSaaaeaacqWGqbaucqGGOaakcqWGubavcqGH9aqpcqaIXaqmcqGGPaqkaeaacqWGqbaucqGGOaakcqWGubavcqGH9aqpcqaIWaamcqGGPaqkaaGaeiilaWcabaGaeyypa0ZaaSaaaeaaiiGacqWFZoWzcqWF7oaBaeaacqaIXaqmcqGHsislcqWFZoWzcqWF7oaBaaGaeiOla4caaaaa@466D@

The prior probability of a sequence labeled with class *C *= 1 not having a site is referred to as *q*, and is

q=Pr(C=1|T=0)=γ(1−λ)1−γλ.
 MathType@MTEF@5@5@+=feaafiart1ev1aaatCvAUfKttLearuWrP9MDH5MBPbIqV92AaeXatLxBI9gBaebbnrfifHhDYfgasaacH8akY=wiFfYdH8Gipec8Eeeu0xXdbba9frFj0=OqFfea0dXdd9vqai=hGuQ8kuc9pgc9s8qqaq=dirpe0xb9q8qiLsFr0=vr0=vr0dc8meaabaqaciaacaGaaeqabaqabeGadaaakeaafaqadeGabaaabaGaemyCaeNaeyypa0dcbiGae8huaaLae8NCaiNaeiikaGIaem4qamKaeyypa0JaeGymaeJaeiiFaWNaemivaqLaeyypa0JaeGimaaJaeiykaKcabaGaeyypa0ZaaSaaaeaaiiGacqGFZoWzcqGGOaakcqaIXaqmcqGHsislcqGF7oaBcqGGPaqkaeaacqaIXaqmcqGHsislcqGFZoWzcqGF7oaBaaGaeiOla4caaaaa@4931@

The probability of a sequence, given that it does not contain a site, is the product of the probabilities for each letter in the sequence according to *θ*_*B*_,

Pr(X|T=0,θ)=∏i=1LθB[Xi].
 MathType@MTEF@5@5@+=feaafiart1ev1aaatCvAUfKttLearuWrP9MDH5MBPbIqV92AaeXatLxBI9gBaebbnrfifHhDYfgasaacH8akY=wiFfYdH8Gipec8Eeeu0xXdbba9frFj0=OqFfea0dXdd9vqai=hGuQ8kuc9pgc9s8qqaq=dirpe0xb9q8qiLsFr0=vr0=vr0dc8meaabaqaciaacaGaaeqabaqabeGadaaakeaaieGacqWFqbaucqWFYbGCcqGGOaakieqacqGFybawcqGG8baFcqWGubavcqGH9aqpcqaIWaamcqGGSaaliiGacqqF4oqCcqGGPaqkcqGH9aqpdaqeWbqaaiab9H7aXnaaBaaaleaacqWGcbGqaeqaaOGaei4waSLaemiwaG1aaSbaaSqaaiabdMgaPbqabaGccqGGDbqxaSqaaiabdMgaPjabg2da9iabigdaXaqaaiabdYeambqdcqGHpis1aOGaeiOla4caaa@4A48@

The DEME motif site model assumes that positions in a site are independent, so the probability of a site is the product of the probabilities of the letters in the site, as specified by *θ*_*M*_. When *T *= 1, the sequence model assumes that sites can, with uniform probability, appear anywhere in a sequence, so the probability of a sequence, given that it contains a site, *Pr*(**X|***T *= 1, *θ*), is

1N(∏i=1LθB[Xi])(∑i=1N∏j=0w−1θM[Xi+j,j]θB[Xi+j]),
 MathType@MTEF@5@5@+=feaafiart1ev1aaatCvAUfKttLearuWrP9MDH5MBPbIqV92AaeXatLxBI9gBaebbnrfifHhDYfgasaacH8akY=wiFfYdH8Gipec8Eeeu0xXdbba9frFj0=OqFfea0dXdd9vqai=hGuQ8kuc9pgc9s8qqaq=dirpe0xb9q8qiLsFr0=vr0=vr0dc8meaabaqaciaacaGaaeqabaqabeGadaaakeaadaWcaaqaaiabigdaXaqaaiabd6eaobaadaqadaqaamaarahabaacciGae8hUde3aaSbaaSqaaiabdkeacbqabaGccqGGBbWwieqacqGFybawdaWgaaWcbaGaemyAaKgabeaakiabc2faDbWcbaGaemyAaKMaeyypa0JaeGymaedabaGaemitaWeaniabg+GivdaakiaawIcacaGLPaaadaqadaqaamaaqahabaWaaebCaeaadaWcaaqaaiab=H7aXnaaBaaaleaacqWGnbqtaeqaaOGaei4waSLaemiwaG1aaSbaaSqaaiabdMgaPjabgUcaRiabdQgaQbqabaGccqGGSaalcqWGQbGAcqGGDbqxaeaacqWF4oqCdaWgaaWcbaGaemOqaieabeaakiabcUfaBjabdIfaynaaBaaaleaacqWGPbqAcqGHRaWkcqWGQbGAaeqaaOGaeiyxa0faaaWcbaGaemOAaOMaeyypa0JaeGimaadabaGaem4DaCNaeyOeI0IaeGymaedaniabg+GivdaaleaacqWGPbqAcqGH9aqpcqaIXaqmaeaacqWGobGta0GaeyyeIuoaaOGaayjkaiaawMcaaiabcYcaSaaa@68B4@

which is equal to

*Pr*(**X**|*T *= 0, *θ*)·*μ*,

where *μ *is the mean of the *odds *of a length-*w *substring in **X **being a site vs. a non-site. That is,

μ=1N∑i=1N∏j=0w−1θM[Xi+j,j]θB[Xi+j].
 MathType@MTEF@5@5@+=feaafiart1ev1aaatCvAUfKttLearuWrP9MDH5MBPbIqV92AaeXatLxBI9gBaebbnrfifHhDYfgasaacH8akY=wiFfYdH8Gipec8Eeeu0xXdbba9frFj0=OqFfea0dXdd9vqai=hGuQ8kuc9pgc9s8qqaq=dirpe0xb9q8qiLsFr0=vr0=vr0dc8meaabaqaciaacaGaaeqabaqabeGadaaakeaaiiGacqWF8oqBcqGH9aqpdaWcaaqaaiabigdaXaqaaiabd6eaobaadaaeWbqaamaarahabaWaaSaaaeaacqWF4oqCdaWgaaWcbaGaemyta0eabeaakiabcUfaBjabdIfaynaaBaaaleaacqWGPbqAcqGHRaWkcqWGQbGAaeqaaOGaeiilaWIaemOAaOMaeiyxa0fabaGae8hUde3aaSbaaSqaaiabdkeacbqabaGccqGGBbWwcqWGybawdaWgaaWcbaGaemyAaKMaey4kaSIaemOAaOgabeaakiabc2faDbaaaSqaaiabdQgaQjabg2da9iabicdaWaqaaiabdEha3jabgkHiTiabigdaXaqdcqGHpis1aaWcbaGaemyAaKMaeyypa0JaeGymaedabaGaemOta4eaniabggHiLdGccqGGUaGlaaa@596D@

#### Objective function

The objective function used by DEME is the conditional log likelihood of the class labels (*C*), given the sequence dataset (*D*) and the data model parameters (*θ*).

F(D,θ)=∑<X,C>∈Dlog⁡P(C|X,θ).
 MathType@MTEF@5@5@+=feaafiart1ev1aaatCvAUfKttLearuWrP9MDH5MBPbIqV92AaeXatLxBI9gBaebbnrfifHhDYfgasaacH8akY=wiFfYdH8Gipec8Eeeu0xXdbba9frFj0=OqFfea0dXdd9vqai=hGuQ8kuc9pgc9s8qqaq=dirpe0xb9q8qiLsFr0=vr0=vr0dc8meaabaqaciaacaGaaeqabaqabeGadaaakeaacqWGgbGrcqGGOaakcqWGebarcqGGSaaliiGacqWF4oqCcqGGPaqkcqGH9aqpdaaeqbqaaiGbcYgaSjabc+gaVjabcEgaNjabdcfaqjabcIcaOiabdoeadjabcYha8Hqabiab+HfayjabcYcaSiab=H7aXjabcMcaPaWcbaGaeyipaWJae4hwaGLaeiilaWIaem4qamKaeyOpa4JaeyicI4SaemiraqeabeqdcqGHris5aOGaeiOla4caaa@4C53@

For each sequence/class pair in the dataset, the key quantities are *F*(**X**, *C*, *θ*) = *P*(*C|***X**, *θ*) which can be shown to be equal to (see Additional File 1)

P(C|X,θ)={(1−q)(1−sig(y)),if C=0,sig(y)+q(1−sig(y)),if C=1.
 MathType@MTEF@5@5@+=feaafiart1ev1aaatCvAUfKttLearuWrP9MDH5MBPbIqV92AaeXatLxBI9gBaebbnrfifHhDYfgasaacH8akY=wiFfYdH8Gipec8Eeeu0xXdbba9frFj0=OqFfea0dXdd9vqai=hGuQ8kuc9pgc9s8qqaq=dirpe0xb9q8qiLsFr0=vr0=vr0dc8meaabaqaciaacaGaaeqabaqabeGadaaakeaacqWGqbaucqGGOaakcqWGdbWqcqGG8baFieqacqWFybawcqGGSaaliiGacqGF4oqCcqGGPaqkcqGH9aqpdaGabeqaauaabaqaciaaaeaacqGGOaakcqaIXaqmcqGHsislcqWGXbqCcqGGPaqkcqGGOaakcqaIXaqmcqGHsislcqqGZbWCcqqGPbqAcqqGNbWzcqGGOaakcqWG5bqEcqGGPaqkcqGGPaqkcqGGSaalaeaacqqGPbqAcqqGMbGzcqqGGaaicqWGdbWqcqGH9aqpcqaIWaamcqGGSaalaeaacqqGZbWCcqqGPbqAcqqGNbWzcqGGOaakcqWG5bqEcqGGPaqkcqGHRaWkcqWGXbqCcqGGOaakcqaIXaqmcqGHsislcqqGZbWCcqqGPbqAcqqGNbWzcqGGOaakcqWG5bqEcqGGPaqkcqGGPaqkcqGGSaalaeaacqqGPbqAcqqGMbGzcqqGGaaicqWGdbWqcqGH9aqpcqaIXaqmcqGGUaGlaaaacaGL7baaaaa@6CD0@

where *q *is given in Eqn. 3, the sigmoid function is defined as

sig(y)=11+e−y,
 MathType@MTEF@5@5@+=feaafiart1ev1aaatCvAUfKttLearuWrP9MDH5MBPbIqV92AaeXatLxBI9gBaebbnrfifHhDYfgasaacH8akY=wiFfYdH8Gipec8Eeeu0xXdbba9frFj0=OqFfea0dXdd9vqai=hGuQ8kuc9pgc9s8qqaq=dirpe0xb9q8qiLsFr0=vr0=vr0dc8meaabaqaciaacaGaaeqabaqabeGadaaakeaacqqGZbWCcqqGPbqAcqqGNbWzcqGGOaakcqWG5bqEcqGGPaqkcqGH9aqpdaWcaaqaaiabigdaXaqaaiabigdaXiabgUcaRiabdwgaLnaaCaaaleqabaGaeyOeI0IaemyEaKhaaaaakiabcYcaSaaa@3C9E@

and *y *is the discriminant function

y=log⁡(Pr(X,T=1|θ)Pr(X,T=0|θ))=log⁡(V⋅μ).
 MathType@MTEF@5@5@+=feaafiart1ev1aaatCvAUfKttLearuWrP9MDH5MBPbIqV92AaeXatLxBI9gBaebbnrfifHhDYfgasaacH8akY=wiFfYdH8Gipec8Eeeu0xXdbba9frFj0=OqFfea0dXdd9vqai=hGuQ8kuc9pgc9s8qqaq=dirpe0xb9q8qiLsFr0=vr0=vr0dc8meaabaqaciaacaGaaeqabaqabeGadaaakeaafaqadeGabaaabaGaemyEaKNaeyypa0JagiiBaWMaei4Ba8Maei4zaC2aaeWaaeaadaWcaaqaaGqaciab=bfaqjab=jhaYjabcIcaOGqabiab+HfayjabcYcaSiabdsfaujabg2da9iabigdaXiabcYha8HGaciab9H7aXjabcMcaPaqaaiab=bfaqjab=jhaYjabcIcaOiab+HfayjabcYcaSiabdsfaujabg2da9iabicdaWiabcYha8jab9H7aXjabcMcaPaaaaiaawIcacaGLPaaaaeaacqGH9aqpcyGGSbaBcqGGVbWBcqGGNbWzcqGGOaakcqWGwbGvcqGHflY1cqqF8oqBcqGGPaqkcqGGUaGlaaaaaa@5B4E@

Substituting the formulas for *μ *(Eqn. 5) and *V *(Eqn. 2) completes the definition of the objective function in terms of the data and model parameters.

These formulas apply to protein sequences, however for DNA, apply only when sites are not allowed on the negative DNA strand. The more general formulas and derivations for DNA sequences allowing sites on either strand are given in the Additional File 1. Details are also given there of the derivation of the partial derivatives of the objective function required by the local search function, conjugate gradient.

#### Global search

DEME applies a two-step approach to identify good starting points for refinement by conjugate gradient. First, substring search is applied to sample all length-*w *substrings from the positive sequences, where *w *is the width of the motif. The best strings identified by substring search are used as starting points for "branching search". Branching search expands the search space by mutating positions in the substrings independently to generate new strings. We use a heuristic implementation of branching using a fixed size heap. During substring search and each iteration of branching, we score strings using the objective function described below and store the top scoring strings in the heap. The strings in the heap are then used for the next iteration of branching. The size of the heap and the number of branching iterations are user inputs to the algorithm.

During substring and branching search, each string is mapped to a corresponding motif PSFM, *θ*_*M*_, and the objective function (Eqn. 6) applied to this PSFM is used as the score for the string. For DNA sequences, we assume that the string is a sample of size one from a motif distribution, and we use mean posterior estimation (MPE) with a uniform Dirichlet prior estimate the parameters, *θ*_*M*_, of the motif model [30]. For protein sequences, we use prior knowledge of amino acid substitution frequencies to map the string to a PSFM, as described below.

The string mapping for DNA sequences to a motif model is given by

θM[a,i]=Na,i+βa1+B for a∈Σ,1≤i≤w,
 MathType@MTEF@5@5@+=feaafiart1ev1aaatCvAUfKttLearuWrP9MDH5MBPbIqV92AaeXatLxBI9gBaebbnrfifHhDYfgasaacH8akY=wiFfYdH8Gipec8Eeeu0xXdbba9frFj0=OqFfea0dXdd9vqai=hGuQ8kuc9pgc9s8qqaq=dirpe0xb9q8qiLsFr0=vr0=vr0dc8meaabaqaciaacaGaaeqabaqabeGadaaakeaaiiGacqWF4oqCdaWgaaWcbaGaemyta0eabeaakiabcUfaBjabdggaHjabcYcaSiabdMgaPjabc2faDjabg2da9maalaaabaGaemOta40aaSbaaSqaaiabdggaHjabcYcaSiabdMgaPbqabaGccqGHRaWkcqWFYoGydaWgaaWcbaGaemyyaegabeaaaOqaaiabigdaXiabgUcaRiabdkeacbaacqqGGaaicqqGMbGzcqqGVbWBcqqGYbGCcqqGGaaicqWGHbqycqGHiiIZcqqHJoWucqGGSaalcqaIXaqmcqGHKjYOcqWGPbqAcqGHKjYOcqWG3bWDcqGGSaalaaa@558D@

where *N*_*a*,*i *_is equal to one if there is a letter *a *at position *i *of the string, or is zero otherwise, the pseudocounts *β*_*a *_are *B*/4 for all letters *a*, and *B *is the "seed prior weight" and is equal to the sum of the pseudocounts. The value of *B *is an input parameter to DEME.

We only use Eqn. 7 to map DNA strings to an initial motif model. To map protein strings, we use probabilities derived from a PAM120 substitution matrix [31]. To create the motif model, we replace each letter in the string with the vector of conditional probabilities of that letter being substituted by each of the other possible letters. If the letter at position *i *of the string is *a*, we set *θ*_*M *_[*b*, *i*] = *Pr*(*b*|*a*) for all *b *∈ Σ. We do this for each position *i *in the string. This mapping assigns high probabilities to amino acid substitutions that are assumed more likely to occur *a priori*.

After the final iteration of branching search, the string with the highest score according to the objective function is converted to a weights matrix, **W**, and input to the local search algorithm, conjugate gradient. To convert from string space to **W **space, the string is mapped to a PSFM, *θ*_*M*_, using one of the two methods just described, and then **W **is set equal to the logarithm of the PSFM.

#### Local search

Local search in DEME, as in the Sharan-Segal algorithm, consists of maximizing the objective function (Eqn. 1) with respect to the model parameters, *θ*, using conjugate gradient optimization. As discussed below, it is necessary to reparameterize the objective function to avoid solutions where *θ*_*M *_and *λ *assume illegal values. No parameterization of *θ*_*B *_or *γ *is required because they are held constant during conjugate gradient. The value of *γ *is estimated by DEME as the ratio of the numbers of positive and negative sequences in the input dataset. The background model *θ*_*B *_is estimated as the frequency of each letter *a *belonging to the alphabet Σ in the dataset.

Each column of *θ*_*M *_is the parameters of a discrete distribution, which must all be non-negative and sum to one. So performing optimization directly on *θ *would require using a constrained optimization method. In order to avoid this, the Sharan-Segal algorithm reparameterized the objective function in terms of a log odds scoring matrix,

Wa,i=log⁡θM[a,i]θB[a],
 MathType@MTEF@5@5@+=feaafiart1ev1aaatCvAUfKttLearuWrP9MDH5MBPbIqV92AaeXatLxBI9gBaebbnrfifHhDYfgasaacH8akY=wiFfYdH8Gipec8Eeeu0xXdbba9frFj0=OqFfea0dXdd9vqai=hGuQ8kuc9pgc9s8qqaq=dirpe0xb9q8qiLsFr0=vr0=vr0dc8meaabaqaciaacaGaaeqabaqabeGadaaakeaaieqacqWFxbWvdaWgaaWcbaGaemyyaeMaeiilaWIaemyAaKgabeaakiabg2da9iGbcYgaSjabc+gaVjabcEgaNnaalaaabaacciGae4hUde3aaSbaaSqaaiabd2eanbqabaGccqGGBbWwcqWGHbqycqGGSaalcqWGPbqAcqGGDbqxaeaacqGF4oqCdaWgaaWcbaGaemOqaieabeaakiabcUfaBjabdggaHjabc2faDbaacqGGSaalaaa@4792@

where **W**_*a*,*i *_is the log odds score of the letter *a *at position *i *in a motif site. It is easy to see that Eqn. 5, the average *odds *score of potential sites in a sequence, can be rewritten using the average *log odds *score of sites. Let

si=∑j=0w−1WXi+j,j=∑j=0w−1log⁡θM[Xi+j,j]θB[Xi+j]
 MathType@MTEF@5@5@+=feaafiart1ev1aaatCvAUfKttLearuWrP9MDH5MBPbIqV92AaeXatLxBI9gBaebbnrfifHhDYfgasaacH8akY=wiFfYdH8Gipec8Eeeu0xXdbba9frFj0=OqFfea0dXdd9vqai=hGuQ8kuc9pgc9s8qqaq=dirpe0xb9q8qiLsFr0=vr0=vr0dc8meaabaqaciaacaGaaeqabaqabeGadaaakeaafaqadeGabaaabaGaem4Cam3aaSbaaSqaaiabdMgaPbqabaGccqGH9aqpdaaeWbqaaGqabiab=DfaxnaaBaaaleaacqWGybawdaWgaaadbaGaemyAaKMaey4kaSIaemOAaOgabeaaliabcYcaSiabdQgaQbqabaaabaGaemOAaOMaeyypa0JaeGimaadabaGaem4DaCNaeyOeI0IaeGymaedaniabggHiLdaakeaacqGH9aqpdaaeWbqaaiGbcYgaSjabc+gaVjabcEgaNnaalaaabaacciGae4hUde3aaSbaaSqaaiabd2eanbqabaGccqGGBbWwcqWGybawdaWgaaWcbaGaemyAaKMaey4kaSIaemOAaOgabeaakiabcYcaSiabdQgaQjabc2faDbqaaiab+H7aXnaaBaaaleaacqWGcbGqaeqaaOGaei4waSLaemiwaG1aaSbaaSqaaiabdMgaPjabgUcaRiabdQgaQbqabaGccqGGDbqxaaaaleaacqWGQbGAcqGH9aqpcqaIWaamaeaacqWG3bWDcqGHsislcqaIXaqma0GaeyyeIuoaaaaaaa@67CA@

be the log odds score of the site starting at position *i *in sequence **X**. Then, *μ *can be rewritten as

μ=1N∑i=1Nexp⁡(si).
 MathType@MTEF@5@5@+=feaafiart1ev1aaatCvAUfKttLearuWrP9MDH5MBPbIqV92AaeXatLxBI9gBaebbnrfifHhDYfgasaacH8akY=wiFfYdH8Gipec8Eeeu0xXdbba9frFj0=OqFfea0dXdd9vqai=hGuQ8kuc9pgc9s8qqaq=dirpe0xb9q8qiLsFr0=vr0=vr0dc8meaabaqaciaacaGaaeqabaqabeGadaaakeaaiiGacqWF8oqBcqGH9aqpdaWcaaqaaiabigdaXaqaaiabd6eaobaadaaeWbqaaiGbcwgaLjabcIha4jabcchaWjabcIcaOiabdohaZnaaBaaaleaacqWGPbqAaeqaaOGaeiykaKcaleaacqWGPbqAcqGH9aqpcqaIXaqmaeaacqWGobGta0GaeyyeIuoakiabc6caUaaa@4220@

The Sharan-Segal algorithm expresses the objective function in terms of **W **and optimizes the reparameterized function using conjugate gradient, allowing the elements of **W **to assume any real values (unconstrained optimization). All other parameters are held fixed.

This approach has two shortcomings. Firstly, not all values of **W **correspond to legal values of the motif model parameters, *θ*_*M*_, so local search can find maxima that do not correspond to valid data models. Therefore, constrained optimization should be applied to prevent **W **from having values that do not map back to valid motif models. This is mainly a technical objection, since the resulting model may still be quite useful. The second shortcoming is that optimizing the objective function with or without the Sharan-Segal reparameterization can over-fit the data when there are few positive sequences since no regularization is applied to the motif parameters (*θ*_*M*_) to account for small sample sizes.

DEME overcomes both of these shortcomings by reparameterizing the objective function differently. Our approach is based on analogy with non-discriminative motif discovery methods where the motif model parameters, *θ*_*M*_, are estimated from observed counts of letters in a local multiple alignment of (putative) motif sites. Mean posterior estimation (MPE) [30] is then used to estimate the true motif parameters, which involves adding pseudocounts based on a Dirichlet (DNA) or mixture of Dirichlet (protein) prior distribution over the parameters of *θ*_*M *_[11]. Doing MPE reduces the chance of over-fitting small sequence sets and brings to bear prior information about likely protein motif columns that captures the tendency of similar amino acids to substitute for each other in motifs.

Rather than defining **W **to be the log odds scoring matrix (Eqn. 8) as done by Sharan-Segal, we think of **W **as being related to the observed counts of letters in a multiple alignment of motif sites. In order for unconstrained optimization over **W **to be appropriate, the entries in **W **must be allowed to assume any real values, so we define a one-way mapping from any **W **to a valid *θ*_*M *_as follows. Firstly, DEME maps **W **to an "observed" PSFM, **f**,

fa,i=exp⁡(Wa,i)Zi,
 MathType@MTEF@5@5@+=feaafiart1ev1aaatCvAUfKttLearuWrP9MDH5MBPbIqV92AaeXatLxBI9gBaebbnrfifHhDYfgasaacH8akY=wiFfYdH8Gipec8Eeeu0xXdbba9frFj0=OqFfea0dXdd9vqai=hGuQ8kuc9pgc9s8qqaq=dirpe0xb9q8qiLsFr0=vr0=vr0dc8meaabaqaciaacaGaaeqabaqabeGadaaakeaacqWGMbGzdaWgaaWcbaGaemyyaeMaeiilaWIaemyAaKgabeaakiabg2da9maalaaabaGagiyzauMaeiiEaGNaeiiCaaNaeiikaGccbeGae83vaC1aaSbaaSqaaiabdggaHjabcYcaSiabdMgaPbqabaGccqGGPaqkaeaacqWGAbGwdaWgaaWcbaGaemyAaKgabeaaaaGccqGGSaalaaa@4160@

where *f*_*a*,*i *_is the observed frequency of a letter *a *in position *i*, and *Z*_*i *_is a normalizing constant for the column *i*,

Zi=∑aexp⁡(Wa,i).
 MathType@MTEF@5@5@+=feaafiart1ev1aaatCvAUfKttLearuWrP9MDH5MBPbIqV92AaeXatLxBI9gBaebbnrfifHhDYfgasaacH8akY=wiFfYdH8Gipec8Eeeu0xXdbba9frFj0=OqFfea0dXdd9vqai=hGuQ8kuc9pgc9s8qqaq=dirpe0xb9q8qiLsFr0=vr0=vr0dc8meaabaqaciaacaGaaeqabaqabeGadaaakeaacqWGAbGwdaWgaaWcbaGaemyAaKgabeaakiabg2da9maaqafabaGagiyzauMaeiiEaGNaeiiCaaNaeiikaGccbeGae83vaC1aaSbaaSqaaiabdggaHjabcYcaSiabdMgaPbqabaGccqGGPaqkaSqaaiabdggaHbqab0GaeyyeIuoakiabc6caUaaa@3FBB@

It is clear that this mapping always gives a legal PSFM. That is, all entries are non-negative and all columns sum to one. It is also one-way, since adding a constant to any column of **W **gives a new matrix that maps to the same PSFM as the old one.

Secondly, DEME converts the observed frequencies (**f**) to "observed counts" (**N**) by multiplying by the number of positive sequences predicted to contain a site (*λN*_*p*_), where *N*_*p *_is the number of positive sequences in the input dataset, yielding

*N*_*a*,*i *_= *λN*_*p*_·*f*_*a*,*i*_.

These first two mapping steps (Eqn. 9 and Eqn. 10) define the analogy between **W **and a multiple alignment of motif sites. The final step of the mapping is to compute the motif parameters, *θ*_*M*_, using MPE [30]. This involves mapping **N **to *θ*_*M *_by adding pseudocounts based on a prior distribution over motif columns, and then normalizing so that each column of *θ*_*M *_sums to one. For DNA sequences, DEME uses a uniform Dirichlet prior. where the pseudocounts, *α*_*a*,*i*_, are all *A*/4 for DNA. The value of *A*, the "motif prior weight", is an input parameter to DEME. For protein sequences, DEME estimates the pseudocounts *α*_*a*,*i *_using a Dirichlet mixture prior [32], where the pseudocounts for column *i *are a function of the observed counts in that column of the alignment [32]. The mean posterior estimate of *θ*_*M *_is given by

θM[a,i]=Na,i+αa,iλNp+Ai,
 MathType@MTEF@5@5@+=feaafiart1ev1aaatCvAUfKttLearuWrP9MDH5MBPbIqV92AaeXatLxBI9gBaebbnrfifHhDYfgasaacH8akY=wiFfYdH8Gipec8Eeeu0xXdbba9frFj0=OqFfea0dXdd9vqai=hGuQ8kuc9pgc9s8qqaq=dirpe0xb9q8qiLsFr0=vr0=vr0dc8meaabaqaciaacaGaaeqabaqabeGadaaakeaaiiGacqWF4oqCdaWgaaWcbaGaemyta0eabeaakiabcUfaBjabdggaHjabcYcaSiabdMgaPjabc2faDjabg2da9maalaaabaGaemOta40aaSbaaSqaaiabdggaHjabcYcaSiabdMgaPbqabaGccqGHRaWkcqWFXoqydaWgaaWcbaGaemyyaeMaeiilaWIaemyAaKgabeaaaOqaaiab=T7aSjabd6eaonaaBaaaleaacqWGWbaCaeqaaOGaey4kaSIaemyqae0aaSbaaSqaaiabdMgaPbqabaaaaOGaeiilaWcaaa@4AC8@

where *A*_*i *_is the sum of the pseudocounts in column *i*.

DEME also reparameterizes the parameter *λ *to prevent it from having illegal values. To do this, DEME introduces a new variable, *W*_*λ*_, which is defined in terms of the original variable, *λ*, as

*λ *= sig(*W*_*λ*_).

This mapping allows unconstrained optimization over *W*_*λ*_, while ensuring that *λ *assumes only values in the range zero to one.

### Discriminative motif discovery problems

We define four synthetic problems for use in evaluating the performance of discriminative motif discovery algorithms such as DEME on artificial data. These problems are intended to represent typical applications for motif discovery in DNA and protein sequences. We create instances of discriminative motif discovery problems by inserting instances of a motif (or motifs) into randomly generated sequences. Motif instances can be generated according to the so-called "FM" model [29], where motifs are represented as a string of width *l *and each instance is a fixed number of mutations, *d*, distant from the motif string. In this work, we introduce a second, novel method of generating motif occurrences based on actual TFBS motifs. Using the JASPAR database, we create artificial motifs, represented as position-specific frequency matrices, by randomly choosing columns from real motifs. Each JASPAR column is represented as a frequency vector. The columns are gathered into a single, novel motif. For each position in the artificial motif, the user can specify the information content range for the column. Only columns in the JASPAR database with information content in the given range are considered when choosing a frequency vector for that position in the motif. Motif instances are then created by sampling from the distribution defined by the motif. More details are given in the Methods section.

1. **Random Negative Problem: **In this problem, the negative sequences contain no useful data, and is included for comparison with the "standard" challenge problem [29]. Instances of the target motif are planted in the positive sequences only. All non-motif positions are randomly generated using a uniform distribution and a 0-order Markov process. This is essentially a non-discriminative motif discovery problem, similar to real applications such as discovering motifs shared by orthologous proteins [33,34], or detecting novel motifs that may be associated genomic imprinting [35].

2. **Decoy Motif Problem: **This problem attempts to model situations where the positive and negative sequences contain one or more motifs in common, but the positive sequences contain an additional, unique motif. A real example of the Decoy motif problem is the search for functional promoter elements in the promoter sequences of co-regulated genes, when polyadenylation sites are also over-represented [36]. To generate an example of this problem, instances of a decoy motif are planted in both positive and negative sequences. The target motif is planted in the positive sequences only. All planted motifs are non-overlapping and are positioned randomly within the sequences. Similar problems have been proposed previously [16,37].

3. **Variant Motif Problem: **This problem models the situation where the positive and negative sequences may be distinguishable only because they contain different variants of the same motif. Biological contexts where variant motifs may occur include sequence motifs that affect structural stability [8]; that is, different variants of a motif in orthologous thermophilic and mesophilic proteins that are related to thermostability (we examine this problem later). Another example is the discovery of variant motifs in the binding domains of the *Haemophilus influenzae *HMW1 and HMW2 proteins which contribute to different binding specificities [38]. To create an instance of this type of problem, the target motif is mutated to generate a variant motif. Instances of the target motif are planted in the positive sequences, and instances of the variant motif are planted in the negative sequences.

4. **Impoverished Negative Problem: **In this problem, the motif is over-represented in the positive sequences and the negative sequences are depleted for the motif. A biological example of the Impoverished Negative problem might be inferring the binding specificity of TFs from ChIP-chip data [5,39], where the bound probe sequences are enriched for the binding motif of the immunoprecipitated TF and the non-binding probe sequences are depleted for the motif (we apply DEME to ChIP-chip data later). For the Impoverished negative problem, the target motif (PWM) is planted in the positive sequences only. The negative set is constructed so that there are no substrings in the negative sequences that match the PWM with a *p*-value less than a specified threshold (see Methods). Similar problems have been proposed previously [15,17] and used for developing algorithms for discriminative motif discovery.

### Evaluating DEME on synthetic datasets

We first evaluate DEME using synthetic datasets. We study its behavior on each of the synthetic problems introduced in the last section. We use these problems to determine the effects of the differences between DEME and the Sharan-Segal algorithm and to compare DEME with a non-discriminative motif discovery algorithm. In particular, we study the effects of and determine default settings the two parameters *A *and *B*, referred to as the "motif prior weight" and "seed prior weight", as well as the size of the heap and number of iterations of branching search to perform. (The functions of these parameters are described in the previous section describing the DEME algorithm.) Because the challenge problems plant motif sites in all positive sequences, DEME is run using the OOPS data model in each of the experiments described in this section.

#### Local search: effect of the Bayesian motif prior

One of the ways our method differs from the Sharan-Segal algorithm is that we incorporate a Bayesian prior on motif columns in order to avoid over-fitting during local search using conjugate gradient. To test the effectiveness of this approach, we measure the accuracy of predicted motifs as a function of the size of the prior. For these tests, we use the the Random Negative Problem, and run only the local search component of DEME, conjugate gradient. This allows us to isolate the contribution of the Bayesian motif prior to the accuracy of motif discovery by DEME.

**Figure 2:**
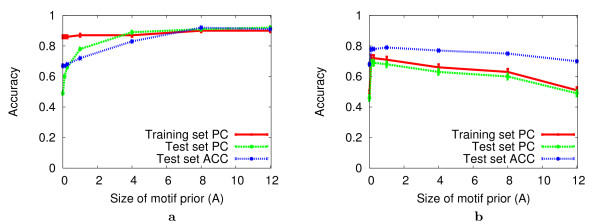
**Effect of the Bayesian motif prior on local search accuracy**. The plot shows the average accuracy of the motif models discovered by *conjugate gradient alone *as a function of *A*, the total pseudocounts applied when deriving the PSFM from **W**. The starting point for conjugate gradient is derived from the consensus sequence for the planted motif using a value of *B *= 0.25. All experiments use the Random Negative Problem and DNA sequences and the OOPS data model. Each data point is the arithmetic mean (± standard error) for 100 independent experiments. Panel **a **shows results using FM motifs and panel **b **shows results using PSFM motifs.

Using the Bayesian motif prior results in a substantial improvement in the accuracy of the motif models learned by conjugate gradient on DNA datasets containing planted FM motifs (Fig. 2a). With FM motifs, values of *A *(refer to Eqn. 11) smaller than eight resulted in less accurate prediction of planted motifs sites (lower training set PC) and much less accurate prediction of motif sites and sequence class (lower test set PC and test set ACC, respectively). This is a strong indication that conjugate gradient over-fits the data when the motifs are FM-like. The extreme case is when *A *= 0, which is similar to the Sharan-Segal use of conjugate gradient. In this case, PC for the training set for FM motifs is 0.86 while PC for the test set is 0.49 and test set ACC is 0.67, compared with training set PC of 0.90, test set PC of 0.91 and ACC of 0.92 when the optimal value of the motif prior is applied. Similarly, training and test set PC for PSFM motifs are 0.49 and 0.46 respectively and test set ACC is 0.68, compared with training set PC of 0.72, test set PC of 0.69 and ACC of 0.78 for the optimal motif prior weight.

The effect of the size of the Bayesian motif prior is very large with FM motifs compared with PSFM motifs. Test set accuracy using PSFM motifs peaks when the motif prior weight is about 1.0 (Fig. 2b). The increase in accuracy compared with using no prior (*A *= 0) is small, however, which indicates that the there is less over-fitting of the data by conjugate gradient when the motif sites are generated by the arguably more natural PSFM model.

In the experiments reported in Fig. 2, the seed for conjugate gradient is the consensus of the target motif; the FM consensus string, or the string with the largest probability given the PSFM. In mapping the seed to a starting point (*θ*_*M*_) for conjugate gradient, we set the seed prior weight, *B*, to 0.25 in Eqn. 7. We also examined values of the seed prior weight in the range [0.01, 4], but found that the value of *B *has very little effect in this setting (data not shown). (As we shall see below, the value of *B *is very important in the context of global search.) Similar results are also obtained when the seed used to initialize *θ*_*M *_for conjugate gradient is Hamming-distance four from the target motif (data not shown). There is, however, an overall decline in the accuracy of the motifs found by conjugate gradient started from these less accurate seeds.

#### Global search: effect of seed prior, heap size and branching

DEME's global search algorithm has several tunable parameters. In this section we use the discriminative motif discovery problems to explore their affect on the accuracy of motifs discovered by DEME. In contrast to the previous section, the experiments here test the entire DEME algorithm, not just the local or global search components. The results, therefore, illustrate the performance of DEME on the various synthetic problems.

**Figure 3:**
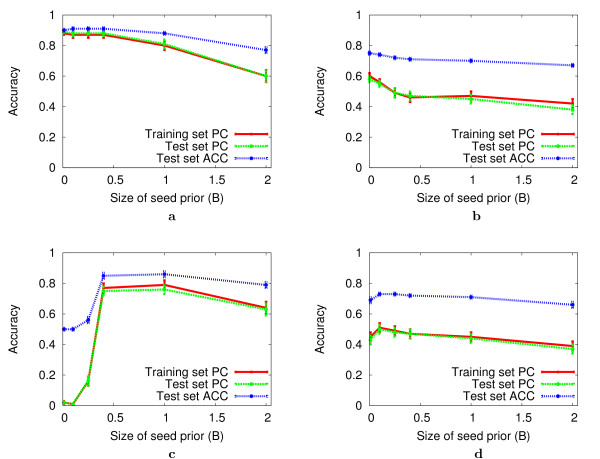
**Effect of the seed prior on DEME accuracy**. The plots show the average accuracy of the motifs discovered by DEME on different synthetic discriminative problems as a function of the size of the seed prior, *B*. The Bayesian motif prior is set to *A *= 4 for FM problems, and *A *= 1 for PSFM problems. All results are for DNA sequences, and DEME uses the OOPS data model in all cases. Each data point is the arithmetic mean ± standard error for 100 independent experiments.

The seed prior weight, *B *(Eqn. 7), directly affects the objective function optimized by DEME during global search. Thus, *B *affects which string motif is chosen by DEME for refinement using local search, strongly influencing the final motif chosen by DEME. Fig. 3 illustrates the effect of *B *on the performance of DEME for the Random Negative and Decoy Motif Problems using both FM and PSFM motifs. In all cases, the training set PC, test set PC and test set ACC measures give similar pictures of DEME's variation in accuracy in response to *B*. DEME performs well with values of *B *smaller than 0.25 on both FM and PSFM motifs in the Random Negative Problem, and with PSFM motifs in the Decoy Motif Problem (Fig. 3a, b, d). However, DEME often fails to discover the true motif in the FM Decoy Motif problem when *B *is less than 0.5 (Fig. 3c). This is due to the global search objective function giving a higher score to sites of the decoy motif compared with the true motif when *B *is small. This effect is absent with the PSFM Decoy Motif Problem, where a very small value of *B*, 0.1, works best. Thus, using *B *= 0.1 seems to be a good compromise, giving optimal or nearly optimal accuracy for all problems except the FM Decoy Motif Problem. Of course, if the motifs in a real dataset are believed to be FM-like, a value of *B *of 0.5 would be appropriate.

The ability of DEME to discover good seeds during global search also depends on the size of the heap and the number of iterations of branching search it performs. In the experiments reported in Fig. 3, the heap size is 250 and the number of branching iterations is six. These were chosen by repeating the experiments in the figure using all combinations of heap size, *H*, and number of branching iterations, *I*, chosen from the sets using all combinations of *H *and *I *where *H *∈ {0, 2, 4, 8, 16, 32, 64, 128, 256} *I *∈ {0, 1, 2, 3, 4, 5, 6, 7, 8, 9, 10}. With FM motifs, the accuracy of the motifs discovered by DEME increases with heap size, where there is only a small improvement in performance when the heap size is larger than 128. For heap sizes of 256, we find that performance plateaus after five iterations of branching. For the PSFM motif problem, branching does not have a large effect on performance (data not shown). Therefore, we use six iterations of branching using a heap size of 250 for all subsequent experiments.

### Performance comparison with non-discriminative motif discovery

**Figure 4:**
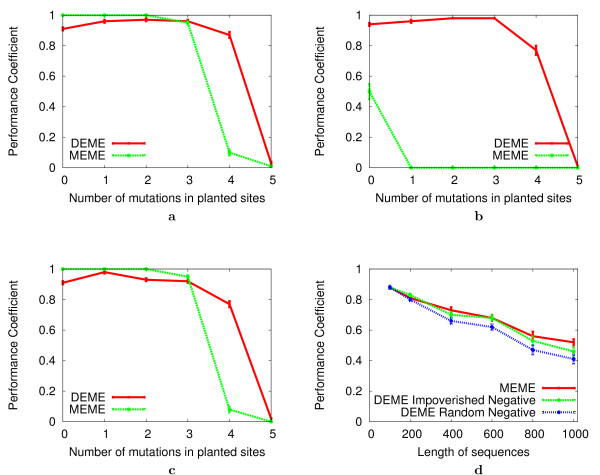
**Comparison of DEME and MEME on synthetic problems**. Each plot shows the accuracy of predicted motifs as measured by the training set PC. Each data point represents the mean (± standard error) PC on 100 independent instantiations of the given problem. Panel **a **shows results on the FM Random Negative Problem. Panel **b **shows results for the FM Decoy Motif Problem with zero mutations in the occurrences of the decoy motif as a function of the number of mutations in the target motif sites. Panel **c **shows results on the FM Variant Motif Problem. The variant motif is Hamming distance four from the target motif and planted instances of the target and variant motifs contain the same number of mutations. Panel **d **shows results on the width-10 PSFM Impoverished Negative Problem (and width-10 PSFM Random Negative Problem for comparison) as a function of the length of the sequences. In all tests, DEME is run using a positive and negative training set, while MEME is applied to the positive training set only.

The FM Random Negative Problem is essentially identical to the well-studied FM Challenge problem [29] for non-discriminative motif discovery. The performance of DEME on this problem for various numbers of mutations in the planted motif of width 15 is shown in Fig. 4a. For comparison, we show the performance of MEME on the just the positive sequences. DEME effectively discovers planted sites that contain up to four mutations, whereas MEME fails to discover planted sites that contain more than three mutations. The performance of DEME on the (15, 4) FM Random Negative problem (see Methods section) is comparable to the performance reported for PROJECTION [40] (PC 0.93) and WINNOWER [29] (PC 0.92). These algorithms were specifically designed to solve the FM Challenge problem, as was the branching search algorithm, on which DEME's global search algorithm is based. The performance of both DEME and PROJECTION declines significantly on the (15, 5) problem, where the performance coefficient for these algorithms is 0.03 and 0.018 respectively. It is not surprising that the performance of these algorithms are poor on the (15, 5) problem, since it is expected that the positive dataset contains spurious motifs that are as "strong" as the planted motif [40].

The Decoy Motif Problem is specifically designed to show the ability of discriminative motif discovery algorithms to find motifs that discriminate between the positive and negative sequences when the two groups of sequences share common motifs. Fig. 4b shows the performance of DEME and MEME on the FM Decoy Motif Problem. In this experiment, one exact copy of a length-15 string (i.e., the decoy motif) is planted in each of the positive and negative sequences. The results show that DEME effectively discovers the target motif in the presence of the decoy, whereas the non-discriminative MEME algorithm does not. DEME's performance on the Decoy Motif Problem is similar to the simpler Random Negative Problem when the planted FM motif occurrences contain up to three mutations, and is slightly poorer when there are four mutations. MEME finds the decoy motif 50% of the time when the target motif sites have the same number of mutations (zero) as the decoy motif. MEME almost always finds the decoy motif if the target sites have more mutations-one or more (data not shown).

The Variant Motif Problem examines the ability of discriminative motif discovery algorithms to find motifs in the positive sequences when there is a variant of the motif in the negative sequences. Fig. 4c shows the performance of DEME and MEME on the FM Variant Motif Problem. In this experiment, the variant motif was generated by mutating exactly four positions in the length-15 target motif. Instances of the target motif are planted in the positive sequences while instances of the variant motif are planted in the negative sequences. The same number of positions in the target and variant motifs are mutated to generate the planted motif instances. The results show that the performance of DEME on the Variant Motif Problem is similar to the performance of DEME on the Random Negative Problem, suggesting that DEME is sensitive to subtle differences between the positive and negative sequences. Since MEME is trained using the positive set only, the performance of MEME on this problem is the same as for the Random Negative Problem.

The Impoverished Negative Problem is targeted at illustrating the ability of discriminative motif finders to find motifs that are over-represented in the positive dataset *relative *to the negative dataset. Fig. 4d shows that DEME does not outperform MEME on the PSFM Impoverished Negative Problem when the negative dataset has the same number of sequences as the positive dataset (twenty). DEME performs slightly worse than MEME when the sequences are long (1000 nt). For comparison, Fig. 4d also shows the performance of DEME on the same positive datasets when the negative datasets contain random sequences (PSFM Random Negative Problem). In this case, DEME's performance is considerably worse, showing that it is benefiting from the relative impoverishment in motif sites of the negative dataset in the PSFM Impoverished Negative Problem. It is evident that the choice of negative set has a significant effect on the performance of DEME, where the best performance is achieved using an impoverished negative set.

### Evaluating DEME on biological datasets

In this section, we evaluate the ability of DEME to discover motifs in real biological datasets. We test on both DNA and protein datasets, and compare with using a non-discriminative motif discovery algorithm. Because real biological datasets often are "noisy", DEME is run using the NOOPS data model in each of the experiments described in this section.

#### Discovery of yeast transcription factor binding motifs

We evaluate the ability of DEME to discover yeast transcription factor binding motifs from the ChIP-chip data reported in [5]. For each experiment, we define the positive training set as the set of probe sequences found to bind the immunoprecipitated transcription factor. For each positive training set, we run two independent experiments. The first experiment uses random negative sequences, where sequences of the negative set are generated by shuffling the letters in the sequences of the positive set. The second experiment uses randomly selected non-binding probe sequences as the negative set (i.e., probe sequences found not to bind the transcription factor under any of the conditions studied by Harbison *et al*. [5]). The positive and negative set contain the same number of sequences when shuffled sequences are used as the negative set, whereas the negative set contains twice as many sequences as the positive set when non-binding probe sequences are used as negative examples. For each experiment we compare the discovered motif to an experimentally determined reference motif for the corresponding transcription factor.

For each experiment, we apply the default value for the motif prior weight and seed prior weight (*A *= 0.25 and *B *= 0.1 respectively). The NOOPS model was applied and both DNA strands were searched. In this experiment, it is assumed that the motif width is known *a priori *and, therefore, the motif width specified to DEME is the width of the corresponding reference motif.

For comparison, MEME (version 3.5.4beta) was also applied to each positive dataset. MEME was run using the ZOOPS sequence model and both DNA strands were searched. MEME was run twice; once using a 0-order background model and once using a fifth-order background model of yeast intergenic regions. Only one motif was reported by each run of MEME and DEME.

We found that DEME is effective for discovering transcription factor binding motifs from ChIP-chip data (refer to Tab. 1). When non-binding probe sequences are used as the negative set, DEME discovers the binding motif for 13 transcription factors. In contrast, when random negative sequences are used (i.e., shuffled positive sequences), DEME discovers the binding motif for only three transcription factors. When random negative sequences are used as the negative set, DEME frequently discovers poly(A) and poly(T) motifs, which are known to be common in yeast intergenic regions [41]. This result suggests that it is important to select appropriate negative sequences according to the hypothesis being tested.

**Table 1:** Summary of the transcription factor binding motifs discovered by DEME and MEME

*Transcription Factor*	DEME		MEME	
	(*R*)	(*NBP*)	(*0-order*)	(*5th-order*)
ABF1		x	x	x
GAL4		x		
GCN4		x	x	x
GCR1			x	x
HAP4			x	x
HSF1		x		x
MBP1	x	x	x	x
MCM1	x	x	x	x
MSN2		x		x
PHO4		x		x
RAP1		x	x	x
REB1	x	x	x	x
SIP4		x		
STE12		x		x
SWI4		x		x

*Total*	3	13	8	13

The first column is the transcription factor name followed by the results for DEME and MEME. DEME is run using randomly shuffled positive sequences (R) or non-binding probes (NBP) as negative sequences. MEME is run using a 0-order or a 5th-order background model. An 'x' indicates a match between the discovered motif and the reference motif. Results are shown for transcription factors where the reference motif was discovered in at least one DEME or MEME experiment. The last row shows the total number of motifs discovered by each algorithm (out of 25 transcription factors).

Using an informative negative sequence set, DEME can outperform the non-discriminative motif finder MEME. With a zero-order background model, MEME discovers the binding motif for only eight transcription factors. However, MEME performs just as well as DEME when provided with a fifth-order background model based on all yeast intergenic regions, In that case, MEME discovers the reference motif for 13 transcription factors (Tab. 1).

It should be noted that our results for the MEME experiments (using a fifth-order background model) do not reproduce the results of Harbison *et al*. [5] exactly. That is, compared to the results published in [5], we report that MEME discovers the binding motifs for an additional two transcription factors (GCR1 and PHO4). Since MEME was run using the same parameters, it is likely that this difference is attributed to different distance metrics used to detect a match between the discovered motif and the reference motif. In our experiment, the consensus of the discovered GCR1 motif is "*CCAGCTTCC*" (*E*-value of 0.002 for the alignment shifted two positions). The published binding specificity for GCR1 is "*GGCTTCCWC*". Similarly, the consensus of the PHO4 motif discovered using MEME is "*ACCCACCTTGTC*" (*E*-value of 0.023), where the PHO4 consensus is "*NNVCACGTRBGN*". The strong statistical support reported by TOMTOM and the similarity between the discovered and reference consensus sequence suggests that MEME has discovered the GCR1 and PHO4 motifs.

The ability of DEME to discover transcription factor binding motifs from ChIP-chip data is comparable to MEME and to other motif discovery algorithms [5]. In combination, both MEME and DEME discover the binding motifs for 16 transcription factors. The binding motifs for the remaining nine transcription factors were also not discovered by any of the six (non-discriminative) motif discovery algorithms tested by Harbison *et al*. [5].

#### Discovery of motifs in thermophilic and mesophilic proteins

As another illustration of the usefulness of DEME, we applied it to the problem of discovering motifs that distinguish between orthologous proteins in thermophilic and mesophilic organisms. A lack of novel thermophilic proteins was observed by La *et al*. [8] which suggests that conserved mutations between orthologous mesophilic and thermophilic proteins might be related to increased thermostability. La *et al*. [8] applied MEME, a non-discriminative motif discovery algorithm, to discover evolutionary conserved differences that distinguish thermophilic proteins from mesophilic proteins. Although successful, this approach is clearly not optimal when the goal is to find motifs that are specifically responsible, or at least strongly correlated with a biological property such as thermal stability. In such a case, a direct, discriminative motif discovery approach using an algorithm such as DEME seems preferable.

We applied DEME to the TATA-box binding protein dataset reported in La *et al*. [8]. The dataset consists of one TATA-box binding protein from each of eight thermophilic microorganisms, and a total of twelve proteins from two mesophilic microorganisms (see Methods section). La *et al*. [8] used these same two sets of proteins to illustrate a two-step method of finding discriminative protein motifs. They first find motifs using a non-discriminative algorithm (MEME), and then filter the motifs, looking for ones that can discriminate the thermophilic set of proteins from the mesophilic proteins.

To compare DEME with the results of La *et al*. [8], we ran DEME twice, using one of the sets of proteins, thermophilic or mesophilic, as the positive set, and the other as the negative set. For each run of DEME, we created two sequence LOGOs [42], showing the residue preferences of the sites that best match the motif discovered by DEME in the positive and negative sequences, respectively. (These sites are reported in the DEME output.) We ran DEME using its default settings and a motif width of 20. The relative heights of the letters in a LOGO are proportional to the number of times that letter occurs in the aligned sites, and the total heights of the letters equals the information content of the frequencies.

**Figure 5:**
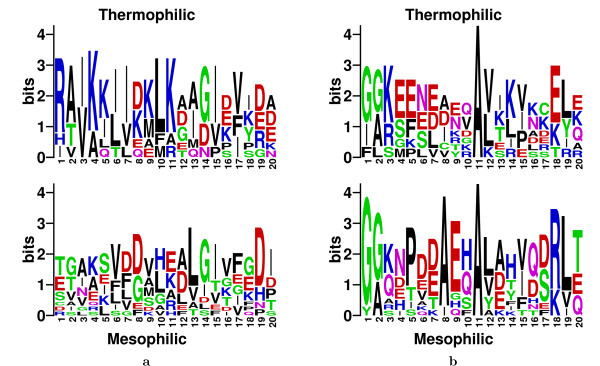
**Discriminative motifs for thermophilic vs. mesophilic TATA-box proteins**. Each column shows the aligned LOGOs from a single experiment. Column **a **shows the motif found by DEME using the thermophilic proteins as the positive set. Column **b **shows the DEME motif when the mesophilic proteins are used as the positive set. In each case, the upper LOGO illustrates the residue preferences in the motif sites reported by DEME in the thermophilic sequences.

DEME finds highly discriminating motifs when either set of sequences (thermophilic or mesophilic) is used as the positive set. Using the thermophilic sequences as the positive set, DEME finds a motif that identifies a region of the TATA-box binding protein that is differentially conserved between the two environments (Fig. 5a). In other words, the motif corresponds to a location in the multiple alignment of all the proteins where there is a strongly conserved but distinct preference for certain residues in the thermophilic compared with the mesophilic proteins. The motif found by DEME motif corresponds to the most highly differentially conserved region in the motif found by La *et al*. [8](see Fig. 6 in La *et al*. [8]). The sites identified by the DEME motif show a very strong preference for the salt-bridge-forming residues arginine (R) and lysine (K) in the thermophilic organisms. These two residues are the most common residue in five out of 20 positions in the thermophilic sites, and are known to enhance thermal stability in proteins. The thermophilic sites also show a very strong preference for isoleucine (I) and valine (V), two residues hypothesized by La *et al*. [8] to promote thermal stability via beta-sheet formation and lower side-chain entropies. These residues (R, K, I and V) are only very weakly preferred in six columns in the sites in the mesophilic sequences; the difference the prevalence of lysine (K) is particularly noticeable.

**Figure 6:**
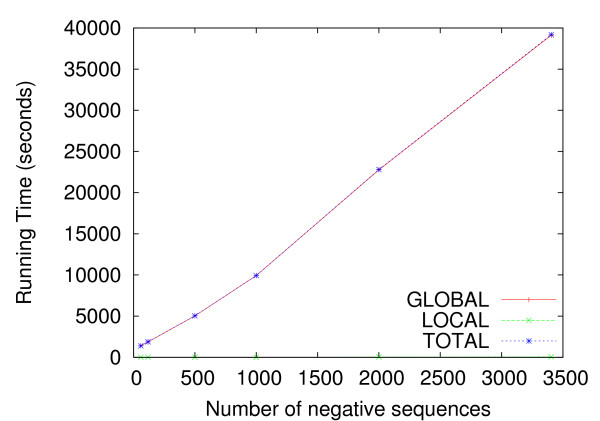
**Running time of the DEME algorithm**. The plot shows the CPU time required by DEME on a typical PC using an typical ChIP-chip (positive) dataset, as a function of the number of non-binding probe sequences as the negative set. The positive set contains 59 probe sequences with an average length of 564 nt that bind GCN4 in rich media (the ChIP-chip datasets compiled by Harbison *et al*. [5] contain on average 40 probe sequences of length 564 nt). Each negative dataset contains randomly selected non-binding probe sequences. The largest negative set studied contains all non-binding probe sequences.

With the roles of the two sequence sets reversed, DEME discovers a different motif (Fig. 5b). The sites in the thermophilic proteins once again show a high preference for salt-bridge-forming residues and the beta-sheet enhancing residues. In this case, the thermophilic sites show a marked preference for glutamate (E), which can form salt-bridges, compared with the mesophilic sites. Glutamate is the most common residue in six out of twenty motif thermophilic site positions. In total, eight thermophilic site positions are dominated by salt-bridge-forming residues (E, K and R) and three columns show a preference for beta-sheet enhancing residues (V and I). The corresponding counts are two (salt-bridge) and one (beta-sheet) dominated columns in the mesophilic sites. These differences show that, in both experiments, DEME has discovered motifs that identify biologically relevant features distinguishing the positive and negative sequence sets.

## Conclusion

Motif discovery in biological sequences is an important but difficult problem. We have shown using real and artificial datasets that DEME is very well suited to finding discriminative motifs in situations where decoy motifs are present in the positive and negative datasets, or when variant motifs are present in the negative dataset. We have also demonstrated that the use of a Bayesian motif prior, made possible by a novel reparameterizing of the Sharan-Segal objective function, can give superior accuracy in DNA motif discovery contexts.

One of the novelties of our work is the development of a general-purpose discriminative motif discovery algorithm for protein as well as DNA. Recognizing the serious problem with over-fitting of motif models in small protein datasets, Lehrach *et al*. [43] used an uninformative, Laplacian prior to regularize the Sharan-Segal model in a special-purpose application (identification of multiple classes of protein-protein interaction motifs.) We believe that DEME is unique among discriminative motif finders in its use of mixture of Dirichlet priors for protein motifs, which previous work on non-discriminative motif discovery with protein datasets [11] has shown to be extremely effective at both reducing over-fitting and at improving the accuracy of motif discovery.

For the yeast TFBS motif discovery problem, DEME performs as well as non-discriminative motif discovery algorithms. Some non-discriminative motif discovery algorithms (such as MEME, evaluated here) can utilize much of the same negative information present in a set of non-binding probes by using a higher-order Markov background sequence model. This probably explains the inability of DEME to outperform MEME on this problem.

The protein motifs discovered by DEME in the mesophilic/thermophilic organism experiment are much more focused and descriptive than the motifs discovered using a non-discriminative approach by La *et al*. [8]. This task is very similar to the Variant Motif Problem, and DEME performs very well on it.

The paper on the ALSE [15] algorithm compares a discriminative PWM-based method (ALSE) to a string-based method (SEEDSEARCH) and to MEME. The test case was discovering TFBSs in promoters of various organisms. Their results indicate that ALSE may be superior to SEEDSEARCH and is definitely superior to MEME. However, in this work MEME uses a 0-order background model, which we have shown (Tab. 1) greatly decreases its performance on this task. It is, therefore, not clear that ALSE is superior to MEME (or DEME) on this task. Because DEME failed to show a marked superiority on the yeast TFBS discovery task (using ChIP-chip data), we did not pursue this question further.

While DEME has many parameters, we have shown that the default parameter settings are effective for discovering biologically significant motifs. The default parameters were determined using the synthetic problems and were found to work very well on the real DNA and protein problems. With the exception of optional parameters (such as specifying that the alphabet is protein), the only parameter that the user must specify is the motif width. The ideal width can be determined by a combination of the user's knowledge of the type of motif being sought and trial-and-error.

Currently, DEME is not optimised for speed. The time complexity of substring search is, as currently implemented in DEME, is

*O*(*N*^+^·*w*·(*N*^+ ^+ *N*^-^)),

where *N*^+ ^and *N*^- ^are the number of length *w *substrings in the sequences in the positive and negative datasets, respectively. The quadratic dependency on the size of the positive sequence set could be reduced to a linear dependency using dynamic programming techniques similar to those used by MEME. The time complexity for pattern branching is

*O*(*I*·*H*·*w*^2^·(|*Σ*| - 1)·(*N*^+ ^+ *N*^-^)),

where *I *is the number of branching iterations and *H *is the heap size. The quadratic dependency on motif width could be reduced to a linear dependency using dynamic programming. The time complexity for local search is

*O*(*w*·(*N*^+ ^+ *N*^-^))

per iteration of conjugate gradient. It is not clear at this point how to improve on the time complexity of local search, but, fortunately, it is only linear in the size of the sequences and the motif width.

The running time for DEME on the ChIP-chip data reported here ranges from 16.9 seconds for the smallest dataset (five positive and ten negative sequences; a total of about 9000 bp (base-pairs) using a motif width of six) to three hours for the largest dataset (178 positive and 356 negative sequences; a total of 270000 bp, using a motif width of fifteen). Here, DEME is run on a processor with a 2.6 GHz CPU and 4 GB memory.

For many applications, such as ChIP-chip datasets, there is an excess of negative sequences. We used a typical ChIP-chip dataset to examine the effect of the number of negative sequences on the running time of DEME. The positive set contained 59 probe sequences with an average length of 564 bp that GCN4 binds (the average ChIP-chip dataset compiled by Harbison *et al*. [5] contains an average of 40 sequences of length 545 bp). We found that the running time of DEME increases linearly as the number of negative sequences increases (data not shown), as we would expect from the complexity calculations above (Fig. 6). The running time of DEME is dominated by global search, where for the datasets examined here, less than 0.05% of the CPU time is used in local search. Therefore, the efficiency of DEME can be greatly improved by optimising the global search algorithm.

Currently, DEME can only find one motif in a given dataset. In order to find multiple motifs, it could be extended in a fashion analogous to the MEME algorithm to find multiple motifs [24]. This can be done by probabilistically "erasing" the predicted sites of a discovered motif, and then repeating the global and local search steps of DEME to discover subsequent motifs. Another possible future enhancement would be having DEME discover the "optimum" motif width.

## Methods

### Performance measures

We use Performance Coefficient (PC) [29] to measure how well the planted sites are discovered at the character level. PC is defined as

PC=|K∩P||K∪P|,
 MathType@MTEF@5@5@+=feaafiart1ev1aaatCvAUfKttLearuWrP9MDH5MBPbIqV92AaeXatLxBI9gBaebbnrfifHhDYfgasaacH8akY=wiFfYdH8Gipec8Eeeu0xXdbba9frFj0=OqFfea0dXdd9vqai=hGuQ8kuc9pgc9s8qqaq=dirpe0xb9q8qiLsFr0=vr0=vr0dc8meaabaqaciaacaGaaeqabaqabeGadaaakeaacqWGqbaucqWGdbWqcqGH9aqpdaWcaaqaamaaemaabaGaem4saSKaeSykIKKaemiuaafacaGLhWUaayjcSdaabaWaaqWaaeaacqWGlbWscqWIQisvcqWGqbauaiaawEa7caGLiWoaaaGaeiilaWcaaa@3E05@

where *K *is the set of known motif positions and *P *is the set of predicted positions. PC is computed on the positive sequences only. When using the NOOPS data model, we predict that a positive sequence contains a site if the probability of the positive class label is greater than 0.5. That is, if *C *= 1 and *P*(*C *= 1|**X**, *θ*) > 0.5. We use as the predicted site the substring in position *i *of sequence **X **with the highest value of the log odds score, *s*_*i *_(Eqn. 8).

We also measure how well the discovered motifs discriminate positive sequences from negative sequences using a set of independent labeled test sequences. The test sets are generated using the same target motif and base distribution as the corresponding training set. The test sets contain 20 sequences of length-600 bases. To measure the discriminative ability of the discovered motif, we use classification accuracy (ACC) which is defined as

ACC=TP+TNP+N
 MathType@MTEF@5@5@+=feaafiart1ev1aaatCvAUfKttLearuWrP9MDH5MBPbIqV92AaeXatLxBI9gBaebbnrfifHhDYfgasaacH8akY=wiFfYdH8Gipec8Eeeu0xXdbba9frFj0=OqFfea0dXdd9vqai=hGuQ8kuc9pgc9s8qqaq=dirpe0xb9q8qiLsFr0=vr0=vr0dc8meaabaqaciaacaGaaeqabaqabeGadaaakeaacqWGbbqqcqWGdbWqcqWGdbWqcqGH9aqpdaWcaaqaaiabdsfaujabdcfaqjabgUcaRiabdsfaujabd6eaobqaaiabdcfaqjabgUcaRiabd6eaobaaaaa@39AD@

where *TP *is the number of true positive predictions, *TN *is the number of true negative predictions, *P *and *N *are the number of positive and negative sequences respectively. A prediction is a true positive if the sequence **X **belongs to the positive class (*C *= 1) and *P*(*C *= 1|**X**, *θ*) > 0.5. Similarly, a true negative is a sequence with the class label *C *= 0 and *P*(*C *= 0|**X**, *θ*) > 0.5. Unlike performance coefficient, which is measured at the level of predicted sites, classification accuracy is measured at the sequence level.

To examine the performance of DEME, we ran 100 independent instantiations of each synthetic problem and compute the mean PC and ACC. For comparison, MEME (version 3.5.4beta) was also applied to the positive training sets and the mean PC was computed. For each MEME experiment, the alphabet was set to DNA, the motif width was set to the width of the generative model and only the top scoring motif was reported. In all cases, the MEME sequence model is set to OOPS. Default settings are applied for all other MEME parameters (unless specified otherwise).

### Generating positive and negative sequences

For each experiment using synthetic data, the positive and negative sets contain 20 sequences of length-600 bases unless specified otherwise. Occurrences of the target motif, called "sites", are planted in a fraction of the positive sequences. All non-motif positions in the positive and negative sequences are randomly generated using a uniform distribution and a 0-order Markov process. The length of the sequence includes the motif sites.

To generate the negative set for the Impoverished Negative Problem, we use a log odds position-specific scoring matrix (PSSM) to represent the motif and use MAST [44] to identify negative sequences that contain matches to the target motif. Any negative sequence containing a substring that matches the target motif with a *p*-value less than 0.001 is discarded. Twenty "impoverished" sequences are then randomly selected as the negative set.

### Generating motif occurrences

Two methods are used to generate motif occurrences. The first applies the "Fixed number of Mutations" (FM) problem described in [29]. The FM model represents a motif as a length-*l *consensus string. Planted motif occurrences contain exactly *d *mismatches from the consensus. This is described as a (*l*, *d*) problem for motif discovery. For experiments using the FM problem, we represent the target motif as a length-15 consensus string which is generated by random sampling from a uniform base distribution. Motif occurrences are generated by mutating exactly *d *positions in the consensus, selected at independently and at random with a uniform probability that a position is mutated. We examine values of *d *in the range [0,5].

The second method we apply to generate motif occurrences is the PSFM method (Whitington and Bailey, in preparation). The motif is represented as a PSFM that is derived from the JASPAR database [45]. To construct a PSFM, matrices from the JASPAR database are split into columns, where each column corresponds to a position in a eukaryotic transcription factor binding motif. The columns are sorted according to information content (IC) and are partitioned into a fixed number of strata of equal size. Columns are randomly sampled from specified strata and are concatenated to form a PSFM. For the PSFM experiments, we use five partitions and columns are sampled from the following strata: 5 5 5 4 4 3 2 2 1 1, where strata 5 contains columns with the highest IC. For the PSFM experiments, we generate length-10 motif instances by sampling from the 4 × 10 PSFM.

### Yeast transcription factor binding site data

We study the ChIP-chip data reported in [5] to examine the ability of DEME to discover transcription factor binding motifs. The ChIP-chip data contains genome wide binding data for 203 yeast transcription factors profiled in 13 environmental conditions. Each dataset contains a set of probe sequences for which the ChIP-chip experiments infer *in vivo *binding of the immunoprecipitated transcription factor. In this study we examine only those transcription factors where there is a corresponding experimentally determined binding profile in SCPD [46] or TRANSFAC [47]. Binding profiles are available for 25 of the 203 transcription factors, providing a total of 59 positive training sets to be examined in this analysis (since 16 of the 25 transcription factors were profiled in multiple environmental conditions).

To determine whether the discovered motif is the binding motif for the immunoprecipitated transcription factor, we compared the discovered motif to a database containing 31 SCPD or TRANSFAC binding profiles. TOMTOM [48] was used to search the database using the discovered motif as the query. Euclidean distance was used to compare the columns of the query motif against each motif in the database and all possible alignments were considered. TOMTOM reports a *p*-value and an *E*-value for each query/target combination. The *E*-value is defined as

*E *= *pN*,

where *p *is the *p*-value and *N *is the number of profiles in the target database. We say that the motif is discovered if the "reference" motif (i.e., the binding profile for the corresponding immunoprecipitated transcription factor) is reported as the best match by TOMTOM with an *E*-value less than 0.05. In addition, the alignment of the reference and discovered motifs must overlap by at least two thirds of the motif length.

### Proteins from thermophilic and mesophilic bacteria

We ran DEME on the sets of TATA-box proteins shown in Tab. 2. These sets of proteins were assembled by La *et al*. [8].

**Table 2:** Sets of orthologous genes used to discover protein motifs

*Thermophiles*		*Mesophiles*	
*organism*	*gene*	*organism*	*gene*
Archaeoglobus fulgidus	AF0373	Saccharomyces cerevisiae	YER148w
Aeropyrum pernix	APE1862	Halobacterium sp. NRC-1	VNG2243G
Methanococcus jannaschii	MJ0507	Halobacterium sp. NRC-1	VNG7031
Methanobacterium thermoautotrophicum	MTH1627	Halobacterium sp. NRC-1	VNG7038
Pyrococcus abyssi	PAB1726	Halobacterium sp. NRC-1	VNG7100
Pyrococcus horikoshii	PH1009	Halobacterium sp. NRC-1	VNG7114
Thermoplasma volcanium	TVN1394	Halobacterium sp. NRC-1	VNG7171
Thermoplasma acidophilum	Ta0199	Halobacterium sp. NRC-1	VNG6037G
		Halobacterium sp. NRC-1	VNG6050G
		Halobacterium sp. NRC-1	VNG6140G
		Halobacterium sp. NRC-1	VNG6438G
		Halobacterium sp. NRC-1	VNG6476G

The TATA-box binding proteins shown in the table were used by La *et al*. [8] to illustrate a discriminative motif discovery approach.

## Authors' contributions

ER did most of the design, implementation and testing of the algorithm, and wrote the initial manuscript draft. TLB proposed the reparameterization of the objective function and the use of pattern-branching for global search, helped design and implement the algorithm, did thermal stability motif experiments, and helped refine the manuscript.

## Supplementary Material

Additional file 1 **Derivations of the DEME Objective Function and Derivatives**. This PDF file (derivation.pdf) describes the derivations of the DEME objective function and its partial derivatives. Click here for file
