# Supplementary material for: Discriminative motif discovery in DNA and protein sequences using the DEME algorithm
Source: BMC Bioinformatics. 2007 Oct 15;8:385. doi: 10.1186/1471-2105-8-385 (PMC2194741; doi:10.1186/1471-2105-8-385)
Supplement: Additional file 1 — Derivations of the DEME Objective Function and Derivatives. This PDF file (derivation.pdf) describes the derivations of the DEME objective function and its partial derivatives. [file 1471-2105-8-385-S1.pdf]

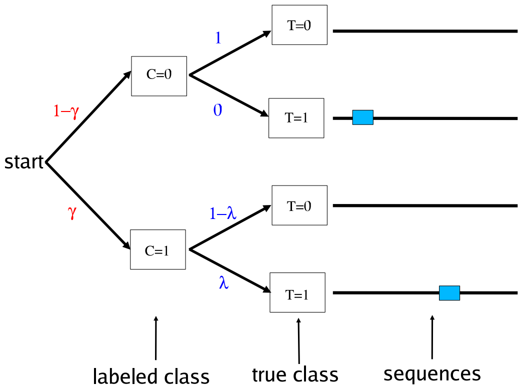

Figure 1: **The** DEME **data model.** Labels on arcs show the probabilities of choosing the labelled class, C, of a sequence, and the true class, T. When $T = 0$, sequences are generated using just the background model, θ_B. When $T = 1$, sequences contain a motif site, generated by motif model θ_M, inserted in random sequence generated by θ_B.

The DEME data model

The input to DEME is a labelled set of sequences, $D = \{< \mathbf{X}, C >\}$, where $\mathbf{X}$ is a sequence and $C \in \{0, 1\}$ is its class. Sequences with $C = 1$ are referred to as "positive" sequences; sequences with $C = 0$ are "negative" sequences [1]. DEME models the sequences in its input set as being generated according to the process illustrated in Fig. 1. First the labelled class, C, is chosen. Then, the true class of the sequence, T, is chosen. Then the sequence is generated. If $T = 0$, a random sequence without a planted motif site is generated using a 0-order Markov process with parameter θ_B. Otherwise, a motif site is generated using the motif PSFM, θ_M, and inserted at a random position in a random sequence generated using θ_B.

The labels on the arrows in Fig. 1 show the probabilities of each step of choosing the labelled and true classes of a sequence. The probability of choosing labelled class $C = 1$ is $Pr(C = 1) = \gamma$. If $C = 0$, the true class will always be $T = 0$, and the sequence will not contain a motif. If $C = 1$ is chosen in the first step, the sequence will have a motif with probability $Pr(T = 1|C = 1) = \lambda$. Thus, λ gives the probability that a sequence labelled $C = 1$ contains a site.

If requested by the user, DEME can fix $\lambda = 1$, in which case all positive sequences are assumed to contain a motif site. This is referred to as the OOPS data model. By default, DEME assumes that some positive sequences may not contain a motif, and will attempt to learn the value of λ. This is referred to as the

NOOPS data model.

In what follows, we will assume that the motif sites have w columns, and that the sequences have length L. For convenience, we introduce variable N to represent the number of possible positions for a motif site, $N = L - w + 1$.

Derivation of the objective function

To find motifs, DEME optimizes an objective function that is the sum of the log conditional probabilities of the class of each sequence,

$$F(D, \theta) = \sum_{<\mathbf{X}, C> \in D} \log Pr(C|\mathbf{X}, \theta), \tag{1}$$

where D is the dataset of labeled sequences $< \mathbf{X}, C >$, $\mathbf{X}$ is a DNA or protein sequence, C is the class label (positive or negative) of the sequence, and θ is the parameters of the data model. In particular, θ consists of a motif model, θ_M, and a background model, θ_B, which describes non-motif sequence, λ, the probability of a positive sequence containing a motif site, and γ, the prior probability of a sequence being labelled positive.

The objective function is a sum of functions on single sequences, so we derive the objective function for a single sequence,

$$F(\mathbf{X}, C, \theta) = \log(Pr(C|\mathbf{X}, \theta)). \tag{2}$$

To do this, we derive a formula for $Pr(C|\mathbf{X}, \theta)$. When $C = 1$, this is equal to

$$\begin{aligned} Pr(C = 1|\mathbf{X}, \theta) &= Pr(C = 1|T = 1, \mathbf{X}, \theta) Pr(T = 1|\mathbf{X}, \theta) \\ &\quad + Pr(C = 1|T = 0, \mathbf{X}, \theta) Pr(T = 0|\mathbf{X}, \theta) \\ &= Pr(T = 1|\mathbf{X}, \theta) + Pr(C = 1|T = 0, \mathbf{X}, \theta) Pr(T = 0|\mathbf{X}, \theta) \\ &= Pr(T = 1|\mathbf{X}, \theta) + q Pr(T = 0|\mathbf{X}, \theta), \end{aligned}$$

since $Pr(C = 1|T = 1, \mathbf{X}, \theta) = 1$, and where, for convenience, we define

$$q = Pr(C = 1|T = 0, \mathbf{X}, \theta).$$

Therefore,

$$Pr(C|\mathbf{X}, \theta) = \begin{cases} (1 - q)(1 - Pr(T = 1|\mathbf{X}, \theta)) & \text{if } C = 0, \\ Pr(T = 1|\mathbf{X}, \theta) + q(1 - Pr(T = 1|\mathbf{X}, \theta)) & \text{if } C = 1. \end{cases}$$

The key quantities to compute are now $Pr(T = 1|\mathbf{X}, \theta)$ and $q = Pr(C = 1|T = 0, \mathbf{X}, \theta)$. We will derive q first.

To derive q, we first observe that the probability of sequence $\mathbf{X}$ given its true class, T, and its labelled class, C, is the same as the probability given only T. As a consequence, we have

$$\begin{aligned}
q &= Pr(C = 1|T = 0, \mathbf{X}, \theta) \\
&= \frac{Pr(\mathbf{X}|C = 1, T = 0, \theta)Pr(C = 1|T = 0, \theta)}{Pr(\mathbf{X}|T = 0, \theta)} \\
&= \frac{Pr(\mathbf{X}|T = 0, \theta)Pr(C = 1|T = 0, \theta)}{Pr(\mathbf{X}|T = 0, \theta)} \\
&= Pr(C = 1|T = 0) \\
&= \frac{Pr(C = 1, T = 0)}{Pr(T = 0)} \\
&= \frac{\gamma(1-\lambda)}{\gamma(1-\lambda) + (1-\gamma)} \\
&= \frac{\gamma(1-\lambda)}{1-\gamma\lambda}. \qquad (3)
\end{aligned}$$

The last relationships can be easily seen from Fig. 1.

We now derive $Pr(T = 1|\mathbf{X}, \theta)$. It is easy to show using Bayes' rule that

$$Pr(T = 1|\mathbf{X}, \theta) = \text{sig}(y), \qquad (4)$$

where

$$y = \log \frac{Pr(\mathbf{X}, T = 1|\theta)}{Pr(\mathbf{X}, T = 0|\theta)}.$$

This allows us to rewrite the class probability in terms of y and q as

$$Pr(C|\mathbf{X}, \theta) = \begin{cases} (1-q)(1-\text{sig}(y)) & \text{if } C = 0, \\ \text{sig}(y) + q(1-\text{sig}(y)) & \text{if } C = 1. \end{cases} \qquad (5)$$

To finish the derivation, we must now derive y in terms of the data model parameters. We write

$$\begin{aligned}
y &= \log \frac{Pr(T = 1|\theta)}{Pr(T = 0|\theta)} \frac{Pr(\mathbf{X}|T = 1, \theta)}{Pr(\mathbf{X}|T = 0, \theta)} \\
&= \log \left(\frac{V}{N} \sum_{i=1}^{N} \prod_{j=0}^{w-1} \frac{\theta_M[X_{i+j}, j]}{\theta_B[X_{i+j}]} \right) \\
&= \log \left(\frac{V}{N} \sum_{i=1}^{N} \exp(s_i) \right) \\
&= \log(V \cdot \mu), \qquad (6)
\end{aligned}$$

where V is the ratio of the prior probabilities of a sequence containing or not containing a motif site,

$$\begin{aligned} V &= \frac{Pr(T=1)}{Pr(T=0)}, \\ &= \frac{\gamma\lambda}{\gamma(1-\lambda)+(1-\gamma)} \\ &= \frac{\gamma\lambda}{1-\gamma\lambda}, \end{aligned} \tag{7}$$

and where we define s_i to be the log odds score of the site starting at position i in sequence $\mathbf{X}$,

$$s_i = \sum_{j=0}^{w-1} \log \frac{\theta_M[X_{i+j}, j]}{\theta_B[X_{i+j}]}, \tag{8}$$

and the variable μ is the mean of the *odds* of a length-w substring in $\mathbf{X}$ being a site vs. a non-site,

$$\mu = \frac{1}{N} \sum_{i=1}^{N} \prod_{j=0}^{w-1} \frac{\theta_M[X_{i+j}, j]}{\theta_B[X_{i+j}]}. \tag{9}$$

We have now written q, V and μ entirely in terms of the model parameters. We now plug the expressions for V (Eqn. 7) and μ (Eqn. 9) into the equation for y (Eqn. 6). We then plug the expression for y into Eqn. 4. Finally, we plug the expressions for y and q into the single-sequence class probability, Eqn. 5, and substitute that into Eqn. 2 to give

$$F(\mathbf{X}, C, \theta) = \begin{cases} \log((1-q)(1-\text{sig}(y))) & \text{if } C = 0, \\ \log(\text{sig}(y) + q(1-\text{sig}(y))) & \text{if } C = 1. \end{cases}$$

The objective function, Eqn. 1, is the sum of these quantities over all points in the dataset, D.

Derivation the partial derivatives of the objective function

We now derive the partial derivatives of the objective function for a single sequence,

$$F(\mathbf{X}, C, \theta) = \log(Pr(C|\mathbf{X}, \theta)),$$

with respect to the reparameterized $\theta = < \mathbf{W}, W_\lambda, \gamma >$. DEME estimates the value of γ from the fraction of positive sequences in the input dataset and treats it thereafter as a constant, so we do not derive the partial derivatives with respect to it.

The relationship of the real model parameters to $\mathbf{W}$ and W_λ is as follows. Firstly, DEME maps $\mathbf{W}$ to an "observed" PSFM, $\mathbf{f}$,

$$f_{a,i} = \frac{\exp(\mathbf{W}_{a,i})}{Z_i}, \tag{10}$$

where $f_{a,i}$ is the observed frequency of a letter a in position i and Z_i is a normalising constant for the column i,

$$Z_i = \sum_a \exp(\mathbf{W}_{a,i}). \tag{11}$$

Secondly, DEME converts the observed frequencies, $\mathbf{f}$, to "observed counts", $\mathbf{N}$, by multiplying by the number of positive sequences predicted to contain a site, λN_p, where N_p is the number of sequences in the positive class ($C = 1$) in the input dataset, giving

$$N_{a,i} = \lambda N_p \cdot f_{a,i}. \tag{12}$$

Finally, θ_M is given by

$$\theta_M[a,i] \quad = \quad \frac{N_{a,i} + \alpha_{a,i}}{\lambda N_p + A_i}, \tag{13}$$

where $\alpha_{a,i}$ are "pseudocounts" for letter a in column i, and A_i is the sum of the pseudocounts in column i. The mapping from W_λ to λ is

$$\lambda \quad = \quad \text{sig}(W_\lambda). \tag{14}$$

Partial derivatives with respect to $\mathbf{W}$

We now derive the partial derivatives of the objective function on a single sequence, $\log(Pr(C|\mathbf{X},\theta))$ with respect to $\mathbf{W}$. We note that that q does not depend on variables $x \in \{W_{a,i}\}$, but that function y does. So, for $x \in \{W_{a,i}\}$, the partial derivative with respect to x is of the single-sequence objective function is

$$\begin{aligned}
\frac{\partial}{\partial x} F(\mathbf{X}, C, \theta) &= \frac{\partial}{\partial x} \log(Pr(C|\mathbf{X},\theta)) \\
&= \frac{1}{Pr(C|\mathbf{X},\theta)} \frac{\partial}{\partial x} Pr(C|\mathbf{X},\theta) \\
&= \frac{1}{Pr(C|\mathbf{X},\theta)} \frac{\partial}{\partial y} Pr(C|\mathbf{X},\theta) \frac{\partial y}{\partial x}. \qquad (15)
\end{aligned}$$

So we have reduced the problem to two tasks–deriving the partial derivative of y with respect to each variable, and deriving the derivative of $Pr(C|\mathbf{X},\theta)$ with respect to y.

We address the second task first. When $C = 0$, the partial derivative of $F(\mathbf{X}, C, \theta)$ with respect to y is

$$\begin{aligned}
\frac{\partial}{\partial y} Pr(C = 0|\mathbf{X},\theta) &= \frac{\partial}{\partial y}(1-q)(1-\text{sig}(y)) \\
&= -(1-q)\text{sig}(y)(1-\text{sig}(y)) \\
&= -\text{sig}(y) Pr(C = 0|\mathbf{X},\theta),
\end{aligned}$$

since

$$\frac{\partial}{\partial x}\text{sig}(x) \quad = \quad \text{sig}(x)(1-\text{sig}(x)).$$

Now, since $Pr(C=1|\mathbf{X},\theta) = 1 - Pr(C=0|\mathbf{X},\theta)$, the derivative when $C=1$ is just the negative of the derivative when $C=0$. Hence, derivative with respect to y for both cases is

$$\frac{\partial}{\partial y}Pr(C|\mathbf{X},\theta) \quad = \quad \begin{cases} -\text{sig}(y)Pr(C=0|\mathbf{X},\theta), & \text{if } C=0, \\ \text{sig}(y)Pr(C=0|\mathbf{X},\theta), & \text{if } C=1. \end{cases} \tag{16}$$

We can now rewrite Eqn. 15 as, for $x \in \{W_{a,i}\}$,

$$\begin{aligned} \frac{\partial}{\partial x}F(\mathbf{X},C,\theta) &= \frac{1}{Pr(C|\mathbf{X},\theta)}\frac{\partial}{\partial y}Pr(C|\mathbf{X},\theta)\frac{\partial y}{\partial x} \\ &= \begin{cases} -\text{sig}(y)\dfrac{\partial y}{\partial x} & \text{if } C=0, \\ \dfrac{\text{sig}(y)}{R}\dfrac{\partial y}{\partial x} & \text{if } C=1, \end{cases} \end{aligned} \tag{17}$$

where R is the ratio of the posterior probabilities of the class labels,

$$R \quad = \quad \frac{Pr(C=1|\mathbf{X},\theta)}{Pr(C=0|\mathbf{X},\theta)}, \tag{18}$$

which can be computed by plugging Eqn. 5 into Eqn. 18.

We now address the second task, that of deriving the partial derivatives of y with respect to the parameters $\mathbf{W}$. Since V is not a function of any of the $W_{a,i}$, and since $y = \log(V\mu)$, we can write

$$\frac{\partial y}{\partial W_{a,i}} \quad = \quad \frac{1}{\mu}\frac{\partial \mu}{\partial W_{a,i}}$$

Since

$$\mu \quad = \quad \frac{1}{N}\sum_{j=1}^{N}\exp(s_j),$$

we have

$$\frac{\partial \mu}{\partial W_{a,i}} \quad = \quad \frac{1}{N}\sum_{j=1}^{N}\exp(s_j)\frac{\partial s_j}{\partial W_{a,i}}. \tag{19}$$

Now we derive the partial derivatives of the score functions, s_j, $j \in [1,\ldots,N]$, with respect to the $W_{a,i}$. Note that the columns of θ_M are independent, so the partial derivative with respect to $W_{a,i}$ of s_j, the log odds score for position j in the sequence, only depends on position $j+i$ in the sequence. Let $\delta_{\{\}}$ be the

Kroenecker delta function, and is equal to one when its subscript is true, zero otherwise. Then the partial derivatives of the log odds score functions s_j with respect to the $W_{a,i}$ are

$$\begin{aligned}
\frac{\partial s_j}{\partial W_{a,i}} &= \frac{\partial}{\partial W_{a,i}} \log\left(\frac{\theta_M[b,i]}{\theta_B[b]}\right) \\
&= \frac{\theta_B[b]}{\theta_M[b,i]} \frac{1}{\theta_B[b]} \frac{\partial \theta_M[b,i]}{\partial W_{a,i}} \\
&= \frac{1}{\theta_M[b,i]} \frac{1}{(N_p + A_i)} \frac{\partial N_{b,i}}{\partial W_{a,i}} \\
&= \frac{\lambda N_p}{N_{b,i} + \alpha_{b,i}} \frac{\partial(\exp(W_{b,i})/Z_i)}{\partial W_{a,i}} \\
&= \frac{\lambda N_p}{N_{b,i} + \alpha_{b,i}} \left[\frac{\exp(W_{b,i})\delta_{\{a=b\}}}{Z_i} + \exp(W_{b,i})(-Z_i^{-2})\frac{\partial Z_i}{\partial W_{a,i}}\right] \\
&= \frac{\lambda N_p}{N_{b,i} + \alpha_{b,i}} \left[\frac{\exp(W_{b,i})\delta_{\{a=b\}}}{Z_i} - \frac{\exp(W_{b,i})\exp(W_{a,i})}{Z_i^2}\right] \\
&= \frac{\lambda N_p}{N_{b,i} + \alpha_{b,i}} \left(f_{b,i}\delta_{\{a=b\}} - f_{b,i} f_{a,i}\right) \\
&= \frac{N_{b,i}}{N_{b,i} + \alpha_{b,i}} \left(\delta_{\{a=b\}} - f_{a,i}\right), \qquad (20)
\end{aligned}$$

where b is the letter at position X_{j+i}. We have made the simplifying assumption that the pseudocounts, $\alpha_{a,i}$, are constant, as they are with DNA sequences using a simple Dirichlet prior. For proteins, the pseudocounts are a function of θ_M, so the above derivation is only approximate.

We can now plug and Eqn. 20 into Eqn. 19 to rewrite the partial derivatives of μ with respect to the $W_{a,i}$ as

$$\begin{aligned}
\frac{\partial \mu}{\partial W_{a,i}} &= \frac{1}{N} \sum_{j=1}^{N} \left(\exp(s_j) \frac{N_{b,i}}{N_{b,i} + \alpha_{b,i}} \left(\delta_{\{a=b\}} - f_{a,i}\right)\right) \\
&= \mu_{a,i}, \qquad (21)
\end{aligned}$$

where the new variables $\mu_{a,i}$ are defined for notational convenience. This allows us to rewrite the partial derivatives of y as with respect to the $W_{a,i}$ as

$$\frac{\partial y}{\partial W_{a,i}} = \frac{\mu_{a,i}}{\mu}. \qquad (22)$$

We can now plug Eqn. 22 into Eqn. 17 to give the final equation for the partial derivatives with respect to the $W_{a,i}$,

$$\frac{\partial F(\mathbf{X}, C, \theta)}{\partial W_{a,i}} = \begin{cases} -\mathrm{sig}(y)\dfrac{\mu_{a,i}}{\mu} & \text{if } C = 0, \\[2ex] \dfrac{\mathrm{sig}(y)}{R}\dfrac{\mu_{a,i}}{\mu} & \text{if } C = 1, \end{cases}$$

where R was defined in Eqn. 18.

Partial derivative with respect to W_λ

In this case, we note that both q and y depend on W_λ. So, when $C = 0$, the partial derivative of $Pr(C|\mathbf{X}, \theta)$ with respect to W_λ is

$$\begin{aligned}
\frac{\partial}{\partial W_\lambda} Pr(C=0|\mathbf{X},\theta) &= \frac{\partial}{\partial W_\lambda}(1-q)(1-\text{sig}(y)) \\
&= \frac{\partial}{\partial \lambda}(1-q)(1-\text{sig}(y))\frac{\partial \lambda}{\partial W_\lambda} \\
&= \left(-(1-\text{sig}(y))\frac{\partial q}{\partial \lambda} - (1-q)\frac{\partial}{\partial \lambda}\text{sig}(y)\right)\frac{\partial \lambda}{\partial W_\lambda} \\
&= -\left((1-\text{sig}(y))\frac{\partial q}{\partial \lambda} + (1-q)\frac{\partial}{\partial \lambda}\text{sig}(y)\right)\frac{\partial \lambda}{\partial W_\lambda}. \qquad (23)
\end{aligned}$$

We first note that since $\lambda = \text{sig}(W_\lambda)$, the derivative of λ with respect to W_λ is

$$\begin{aligned}
\frac{\partial \lambda}{\partial W_\lambda} &= \text{sig}(W_\lambda)(1-\text{sig}W_\lambda) \\
&= \lambda(1-\lambda).
\end{aligned}$$

Referring to the equation for q, Eqn. 3, we see that the partial derivative of q with respect to λ is

$$\begin{aligned}
\frac{\partial q}{\partial \lambda} &= \frac{(1-\gamma\lambda)(-\gamma) - \gamma(1-\lambda)(-\gamma)}{(1-\gamma\lambda)^2} \\
&= \frac{-\gamma + \gamma^2\lambda + \gamma^2 - \gamma^2\lambda}{(1-\gamma\lambda)^2} \\
&= \frac{\gamma(\gamma-1)}{(1-\gamma\lambda)^2}
\end{aligned}$$

Referring to the equation for y, Eqn. 6, and noting that μ is not a function of λ, the derivative of $\text{sig}(y)$ with respect to λ is

$$\begin{aligned}
\frac{\partial}{\partial \lambda}\text{sig}(y) &= \text{sig}(y)(1-\text{sig}(y))\frac{\partial}{\partial \lambda}\log(V\mu) \\
&= \text{sig}(y)(1-\text{sig}(y))\frac{1}{V\mu}\mu\frac{\partial}{\partial \lambda}V \\
&= \frac{\text{sig}(y)(1-\text{sig}(y))}{V}\frac{\partial}{\partial \lambda}\frac{\gamma\lambda}{1-\gamma\lambda} \\
&= \frac{\text{sig}(y)(1-\text{sig}(y))}{V}\frac{(1-\gamma\lambda)\gamma - \gamma\lambda(-\gamma)}{(1-\gamma\lambda)^2} \\
&= \frac{\text{sig}(y)(1-\text{sig}(y))}{V}\frac{\gamma - \gamma^2\lambda + \gamma^2\lambda}{(1-\gamma\lambda)^2} \\
&= \frac{\text{sig}(y)(1-\text{sig}(y))}{V}\frac{\gamma}{(1-\gamma\lambda)^2}
\end{aligned}$$

We can now substitute these equations into Eqn. 23 to yield

$$\frac{\partial}{\partial W_\lambda} Pr(C=0|\mathbf{X},\theta) = -\left((1-\text{sig}(y))\frac{\gamma(\gamma-1)}{(1-\gamma\lambda)^2} + \frac{(1-q)\text{sig}(y)(1-\text{sig}(y))}{V}\frac{\gamma}{(1-\gamma\lambda)^2}\right)\lambda(1-\lambda)$$

$$
\begin{aligned}
&= -\frac{\gamma\lambda(1-\lambda)(1-\text{sig}(y))}{(1-\gamma\lambda)^2}\left((\gamma-1)+\frac{(1-q)\text{sig}(y)}{V}\right) \\
&= -\frac{\lambda q(1-\text{sig}(y))}{1-\gamma\lambda}\left(\gamma-1+\frac{(1-q)\text{sig}(y)}{V}\right) \\
&= \lambda q(1-\text{sig}(y))\left((1-q)-\frac{(1-q)\text{sig}(y)}{\gamma\lambda}\right) \\
&= \lambda q(1-q)(1-\text{sig}(y))\left(1-\frac{\text{sig}(y)}{\gamma\lambda}\right) \\
&= q(1-q)(1-\text{sig}(y))(\lambda-\text{sig}(y)/\gamma). \qquad (24)
\end{aligned}
$$

So, using Eqn. 2, we now complete the derivation of the partial derivative of $F(\mathbf{X}, C, \theta)$ with respect to W_λ as, when $C = 0$

$$
\begin{aligned}
\frac{\partial F(\mathbf{X}, C=0, \theta)}{\partial W_\lambda} &= \frac{q(1-q)(1-\text{sig}(y))(\lambda-\text{sig}(y)/\gamma)}{Pr(C=0|\mathbf{X},\theta)} \\
&= \frac{q(1-q)(1-\text{sig}(y))(\lambda-\text{sig}(y)/\gamma)}{(1-q)(1-\text{sig}(y))} \\
&= q(\lambda-\text{sig}(y)/\gamma).
\end{aligned}
$$

Rearranging Eqn. 5 we note that $Pr(C = 1|\mathbf{X}, \theta) = q(1 - \text{sig}(y)(1 - 1/q))$. Using the fact that the equation for the partial derivative of $Pr(C = 1|\mathbf{X}, \theta)$ is just the negative of Eqn. 24 whe have, for $C = 1$,

$$
\begin{aligned}
\frac{\partial F(\mathbf{X}, C=1, \theta)}{\partial W_\lambda} &= \frac{-q(1-q)(1-\text{sig}(y))(\lambda-\text{sig}(y)/\gamma)}{Pr(C=1|\mathbf{X},\theta)} \\
&= \frac{-q(1-q)(1-\text{sig}(y))(\lambda-\text{sig}(y)/\gamma)}{q(1-\text{sig}(y)(1-1/q))} \\
&= \frac{(1-q)(1-\text{sig}(y))(\lambda-\text{sig}(y)/\gamma)}{\text{sig}(y)(1-1/q)-1}.
\end{aligned}
$$

Therefore, the partial derivative of the objective function with respect to W_λ is

$$
\frac{\partial F(\mathbf{X}, C, \theta)}{\partial W_\lambda} = \begin{cases} q(\lambda-\text{sig}(y)/\gamma) & \text{if } C=0, \\ \dfrac{(1-q)(1-\text{sig}(y))(\lambda-\text{sig}(y)/\gamma)}{\text{sig}(y)(1-1/q)-1} & \text{if } C=1. \end{cases}
$$

Derivations when sites can be on either DNA strand

If the positive sequence model allows sites on either DNA strand (+, positive or −, negative) with equal probability, we write

$$
Pr(\mathbf{X}|T=1,\theta) = \frac{Pr(\mathbf{X}|T=1,S=+,\theta)+Pr(\mathbf{X}|T=1,S=-,\theta)}{2},
$$

where S indicates the strand on which the motif site occurs. The first term in the numerator is identical to the positive class-distribution in the case where sites must be on the positive (given) DNA strand. The second term indicates when the strand occurs on the negative (reverse complement) strand.

For each DNA letter a, we write $\bar{a}$ to indicate its complement. We make the simplifying assumption that the background model is "balanced", that is,

$$\theta_B[a] = \theta_B[\bar{a}] \text{ for all } a \in \Sigma.$$

This leads to the following formula for the case when the motif site is on the negative strand:

$$\begin{aligned} Pr(\mathbf{X}|T=1, S=-, \theta) &= \frac{1}{N}\left(\prod_{i=1}^{L} \theta_B[\overline{X_i}]\right)\left(\sum_{i=1}^{N}\sum_{j=0}^{w-1} \frac{\theta_M[\overline{X_{i+w-1-j}}, j]}{\theta_B[\overline{X_{i+w-1-j}}]}\right) \\ &= Pr(\mathbf{X}|T=0,\theta)\frac{1}{N}\sum_{i=1}^{N}\exp\left(\overline{s_i}\right) \\ &= Pr(\mathbf{X}|T=0,\theta)\bar{\mu}, \end{aligned}$$

where $\overline{s_i}$ is the log odds score on the reverse complement strand corresponding to position i of the positive strand, and $\bar{\mu}$ is the average odds of subsequences in the reverse complement of $\mathbf{X}$ being site vs. non-site.

We can now write the distribution when sites can be on either DNA strand as

$$Pr(\mathbf{X}|T=1,\theta) = Pr(\mathbf{X}|T=0,\theta)\frac{\mu+\bar{\mu}}{2}. \tag{25}$$

It is trivial to show that, in this case,

$$y = \log\left(V \cdot \frac{\mu+\bar{\mu}}{2}\right),$$

and the equation for the objective function on one sequence in terms of y, Eqn. 2, is unchanged.

The derivation of the partial derivatives is also straightforward. The only change is the partial derivatives of y with respect to the elements of $\mathbf{W}$ are now

$$\begin{aligned} \frac{\partial y}{\partial W_{a,i}} &= \frac{1}{\mu+\bar{\mu}} \cdot \frac{\partial(\mu+\bar{\mu})}{\partial W_{a,i}} \\ &= \frac{\mu_{a,i}+\overline{\mu_{a,i}}}{\mu+\bar{\mu}}, \end{aligned}$$

where

$$\begin{aligned} \frac{\partial \bar{\mu}}{\partial W_{a,i}} &= \frac{1}{N}\sum_{j=1}^{N}\left(\exp(\overline{s_j})\delta_{\{\overline{X_{j+w-1-i}}=a\}}\right) \\ &= \overline{\mu_{a,i}}. \end{aligned}$$

This results in the following equations for the partial derivatives when the sites can be on either DNA strand:

$$\frac{\partial F(\mathbf{X}, C, \theta)}{\partial W_{a,i}} = \begin{cases} -\text{sig}(y) \dfrac{\mu_{a,i} + \overline{\mu_{a,i}}}{\mu + \bar{\mu}} & \text{if } C = 0, \\ \dfrac{\text{sig}(y)}{R} \dfrac{\mu_{a,i} + \overline{\mu_{a,i}}}{\mu + \bar{\mu}} & \text{if } C = 1. \end{cases}$$

References

1. Sharan R, Myers EW: **A motif-based framework for recognizing sequence families**. *Bioinformatics* 2005, **21**:I387–I393.
